# Multi-modal imaging probe for assessing the efficiency of stem cell delivery to orthotopic breast tumours†
†Electronic supplementary information (ESI) available. See DOI: 10.1039/d0nr03237a


**DOI:** 10.1039/d0nr03237a

**Published:** 2020-08-04

**Authors:** May Zaw Thin, Helen Allan, Robin Bofinger, Tomas D. Kostelec, Simon Guillaume, John J. Connell, P. Stephen Patrick, Helen C. Hailes, Alethea B. Tabor, Mark F. Lythgoe, Daniel J. Stuckey, Tammy L. Kalber

**Affiliations:** a UCL Centre for Advanced Biomedical Imaging , Division of Medicine , University College London , London , WC1E 6DD , UK . Email: t.kalber@ucl.ac.uk ; Email: may.zawthin@crick.ac.uk; b Department of Chemistry , University College London , 20 , Gordon Street , London , WC1H 0AJ , UK

## Abstract

Stem cells have been utilised as anti-cancer agents due to their ability to home to and integrate within tumours. Methods to augment stem cell homing to tumours are being investigated with the goal of enhancing treatment efficacy. However, it is currently not possible to evaluate both cell localisation and cell viability after engraftment, hindering optimisation of therapy. In this study, luciferase-expressing human adipocyte-derived stem cells (ADSCs) were incubated with Indium-111 radiolabelled iron oxide nanoparticles to produce cells with tri-modal imaging capabilities. ADSCs were administered intravenously (IV) or intracardially (IC) to mice bearing orthotopic breast tumours. Cell fate was monitored using bioluminescence imaging (BLI) as a measure of cell viability, magnetic resonance imaging (MRI) for cell localisation and single photon emission computer tomography (SPECT) for cell quantification. Serial monitoring with multi-modal imaging showed the presence of viable ADSCs within tumours as early as 1-hour post IC injection and the percentage of ADSCs within tumours to be 2-fold higher after IC than IV. Finally, histological analysis was used to validate engraftment of ADSC within tumour tissue. These findings demonstrate that multi-modal imaging can be used to evaluate the efficiency of stem cell delivery to tumours and that IC cell administration is more effective for tumour targeting.

## Abstract 2

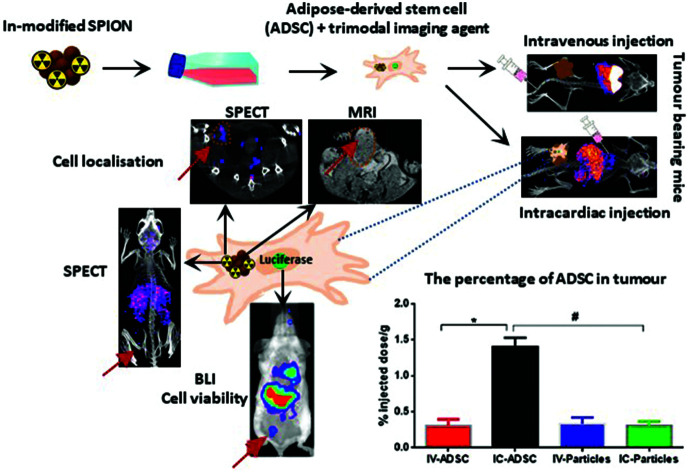
Illustration of adipose-derived stem cells with tri-modal imaging capabilities for evaluating the efficiency of cell delivery to tumours.

## Introduction

Mesenchymal stem cells (MSCs), also called mesenchymal stromal cells, are a promising cell type for regenerative medicine and comprise ∼38% of registered cell therapy clinical trials.^1^ MSCs are also attractive anti-cancer delivery vehicles as they can be readily isolated and expanded from multiple sources; can be genetically manipulated^2^*ex vivo* to express anti-cancer agents such as TRAIL (tumour necrosis factor-related apoptosis induced ligand);^3^,^4^ and show evidence of tumour tropism for targeted delivery of an anti-cancer payload.^5^–^7^

Route of administration has a profound effect on cell distribution and migration.^3^ Intravenous (IV) injection is the most common route for delivering cells, but this leads to the majority becoming trapped within the pulmonary capillary bed immediately after injection, hindering their distribution and migration to distal tissues.^8^–^10^ Alternative injection routes, such as intra-arterial, avoid first-pass to the lungs and could provide a beneficial increase in cell uptake and engraftment in target tissues. Therefore, in order to optimise and evaluate the therapeutic efficacy of a cell therapy, the localisation, retention and viability status of transplanted cells within target organs needs to be assessed.

There are two general approaches in cell labelling for *in vivo* imaging: direct cell labelling and reporter gene imaging.^11^ Direct cell labelling is readily achieved by introducing labelling agents prior to transplantation. These labelling agents are typically internalised by cells and serve as a surrogate measurement of cell location. Reporter gene imaging is achieved by permanent or transient viral transduction or non-viral transfection of non-native DNA for the expression of a specific cell surface receptor, transporter or enzyme that can be utilised to incorporate or activate an imaging probe within the cell.^11^,^12^

Stem cells have been directly labelled with superparamagnetic iron oxide nanoparticles (SPIONs) for magnetic resonance imaging (MRI),^13^–^15^ and utilised in clinical trials to confirm the success or failure of stem cell delivery.^16^–^18^ Although MRI provides excellent spatial resolution of cell localisation in specific organs, quantification is challenging due to the dephasing effect SPIONs have on the surrounding magnetic spins, leading to “blooming” of the signal void and overestimation of cell number.^19^

Another common direct labelling method is Indium-111(^111^In)-oxine for single-photon emission computed tomography (SPECT) imaging, which has been used since the 1970s in the clinic to track white blood cells to areas of inflammation.^20^ This methodology has been applied to tracking stem cells in both preclinical and clinical studies,^9^,^18^,^21^ and has been shown to be a sensitive method of tracking relatively low numbers of cells throughout the body.^22^ The main advantage of SPECT imaging is its ability to semi-quantitatively assess the proportion of cells delivered to target organs relative to the total amount injected (% injected dose).^23^ However, the spatial resolution of SPECT is poor and it is hindered by the dilution of labelling agents upon cell division and the release of radio-label from the cell before or after cell death to resident cells.

Bioluminescence imaging (BLI) is the most frequently used reporter gene for stem cell tracking in small animal studies.^24^–^26^ Since the light emission from BLI is driven by the firefly luciferase gene, there is no background, providing high sensitivity for cell detection. The concept is based on the oxidation of the substrate d-luciferin by the luciferase enzyme in the presence of ATP which results in light emission.^27^ No active process of oxidation can be achieved after cell death and no signal is produced. Although BLI has limited spatial resolution and tissue penetration due to light scattering, it can be used to assess cell viability which is not achievable with direct cell labelling.

Since no single imaging modality can offer structural and quantitative information on cell localisation and cell viability with adequate sensitivity and specificity, there is an urgent need to develop multi-modal imaging probes for cell tracking.^28^ Recent advances in nanochemistry and imaging technologies have facilitated the synthesis of multi-modal imaging probes. These include ^64^Cu–DOTA–SPION conjugates as a PET/MRI imaging agent for tumour imaging^29^ and fluorescent silica coated SPIONs with ^125^Iodine (^125^I) as a SPECT/MRI/fluorescent imaging agent for stem cell tracking.^30^ However, the combination of direct labelling methods only provides extra data on cell localisation, not cell viability.

In this study, a modified SPION with ^111^In was developed as a SPECT/MRI dual imaging agent. This was combined with BLI to acquire comprehensive information on the whole-body distribution and viability of transplanted human adipose derived stem cell (ADSC) following two systemic injection routes; IV injection and intracardiac injection (IC, *via* left ventricular cavity) to compare venous and arterial routes. Since ADSCs have been used as cancer drug delivery tools,^31^ this imaging approach was applied in an orthotopic breast tumour model to compare routes of administration for efficient delivery of stem cells to distal tumours.

## Results and discussion

### Synthesis and analysis of modified SPIONs

FluidMag CT nanoparticles (100 nm) were chosen due to commercial availability and previous evaluation in stem cell labelling.^32^ To increase the indium content of the particles, we attempted to functionalise the outer citric acid polymer matrix with a 1,4,7,10-tetraazacyclododecane-1,4,7,10-tetraacetic acid (DOTA) linker (Fig. S1, see ESI–1† for synthesis and characterisation of the DOTA-linker including ^1^H and ^13^C NMR spectra) as DOTA is known to form thermodynamically stable complexes with ^111^In.^33^ The manipulated SPIONs were examined in deionised water by transmission electron microscopy (TEM) and compared to SPIONs only. Both SPION and modified SPION showed similar morphological structure and aggregation (ESI Fig. S2†). No diameter measurement was performed due to aggregation. Particle aggregation is mainly due to the large size (100 nm) and a multi-domain core of original fluidMAG-CT.^34^,^35^ The hydrodynamic diameter (nm) and zeta potential (mV) of SPIONs alone and modified SPIONs were measured in deionised water at 25 °C using dynamic light scattering (DLS). The modified SPIONs had a decreased hydrodynamic diameter (98.6 ± 0.7 nm) compared to unmodified SPIONs (103.1 ± 0.2 nm). In terms of surface charge, modified SPIONs became more negatively charged (–39.7 ± 0.5 mV) compared to SPION alone (–23.2 ± 0.5 mV). Although there was a change in zeta potential associated with our modified-SPIONs, additional analysis carried out after incubation with InCl_3_ showed that no DOTA-linker coupling had occurred. The changes in surface charge observed are likely to be due to the loss of sodium counter-ions from the citrate groups on the SPION during the coupling reaction.

Despite an increased indium content in the modified particles, measured by inductively coupled plasma atomic emission spectroscopy (ICP-AES), analysis by X-ray photoelectron spectroscopy (XPS) showed indium but no nitrogen content and hence no coordination of the DOTA-linker (ESI Fig. S3†). Infrared spectroscopy analysis of our manipulated nanoparticles provided few insights due to the broad signals present. However, in terms of the presence of DOTA, the data was inconclusive due to overlapping signals of the citrate coating of the SPIONs and the linker. In order to evaluate the success of the coupling reaction we repeated the procedure using a fluorescent dye (cyanine-3 amine). The concentration of the dye in solution, after reaction and magnetic separation of the SPIONs, was measured using UV absorption and it was found that no coupling had occurred. This highlights the importance of full characterisation of nanoparticles, as a number of groups solely use changes in zeta potential and particle size as evidence for desired surface coupling reactions.

### Stability and indium content of modified SPIONs

**Fig. 1:**
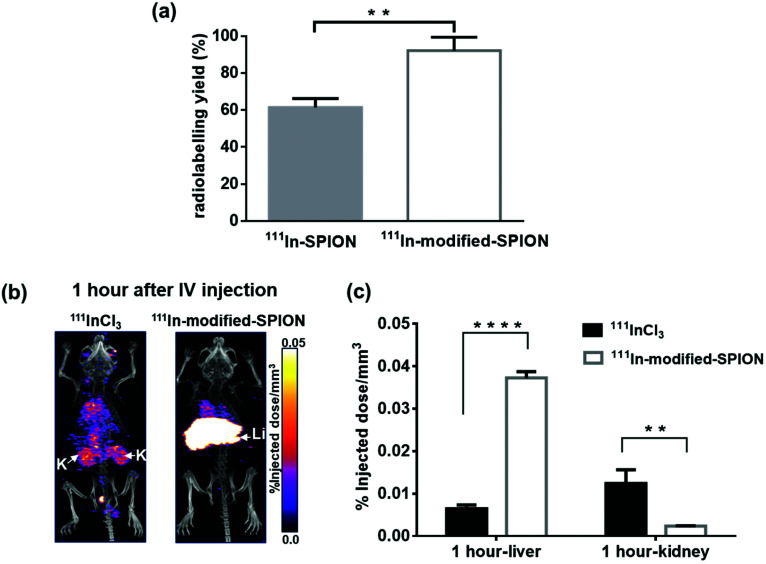
*In vitro* and *in vivo* validation of ^111^In-modified-SPION stability. (a) The % radiolabelling yield of ^111^In-SPION and ^111^In-modified-SPION after 4 hours incubation with ^111^In at 70 °C (** *P* = 0.0035 as measured with unpaired two-tailed *t* test). (b) SPECT/CT images at 1 hour after IV injection of free ^111^InCl_3_ and ^111^In-modified-SPION (labels: Li = Liver, K = Kidney). (c) 3D ROI quantification of SPECT signal in liver and kidney at 1 hour after IV injection calculated as %ID mm^–3^ (in liver, **** *P* < 0.0001 and in kidney, ** *P* = 0.0059 as measured with unpaired two-tailed *t* test). Data are shown as mean ± standard deviation (SD), *n* = 3.

ICP-AES was used to measure both the indium : iron content of SPIONs and the stability of In-SPION and In-modified-SPION in water over 7 days. After 4 hours incubation with InCl_3_ (non-radioactive) at 70 °C, it was found that In-modified-SPION contained just over double the amount of indium compared to In-SPIONs (In : Fe molar ratio of 1 : 102 compared to 1 : 220). Particles were washed after 1, 3 and 7 days to remove any dissociated indium and subjected to ICP-AES. In all cases no erosion of indium content was seen. Thin-layer chromatography (TLC) was used to measure the radiolabelling yield of ^111^In-SPION and ^111^In-modified-SPION. The results showed the radiolabelling yield of ^111^In-modified-SPION to be 92 ± 7%, 1.5-fold higher than ^111^In-SPION (*P* = 0.0035, Fig. 1a). The results observed are likely to be due to the sodium counter-ion of the citrate groups on SPION being replaced during the coupling reaction, leading to greater indium incorporation.

To study *in vivo* stability of ^111^In-modified-SPION complex, mice were injected IV with either free ^111^InCl_3_ or ^111^In-modified-SPION particles and imaged with SPECT/CT serially over 7 days. Since the liver is the known clearance pathway for nanoparticles above ∼9 nm,^36^,^37^ and the kidney is the normal excretion route for free ^111^InCl_3_,^38^ and nanoparticles below ∼9 nm,^37^ 3D regions of interest (ROI) for these organs were created to determine the amount of radioactivity (percent injected dose per mm^3^, (%ID mm^–3^)). At 1-hour after injection, the %ID mm^–3^ in liver was significantly higher in the ^111^In-modified-SPION group compared to free ^111^InCl_3_ (*P* < 0.0001, Fig. 1b and c). Conversely, the %ID mm^–3^ in kidney was significantly lower in the ^111^In-modified-SPION group compared to free ^111^InCl_3_ (*P* = 0.0059, Fig. 1b and c). At days 1, 3 and 7 after injection, the %ID mm^–3^ in liver remained stable in the ^111^In-modified-SPION group with no significant changes in kidney uptake (ESI Fig. S4†). These results demonstrate that ^111^In is stably bound to modified-SPION *in vivo*. Even though there was no DOTA attachment, the modification process increased indium content and offered stable binding to particles. Hence, the modified-SPONs were taken forward for further *in vivo* studies.

### Labelling of ADSCs with ^111^In-modified-SPION and the effect on cell function

**Fig. 2:**
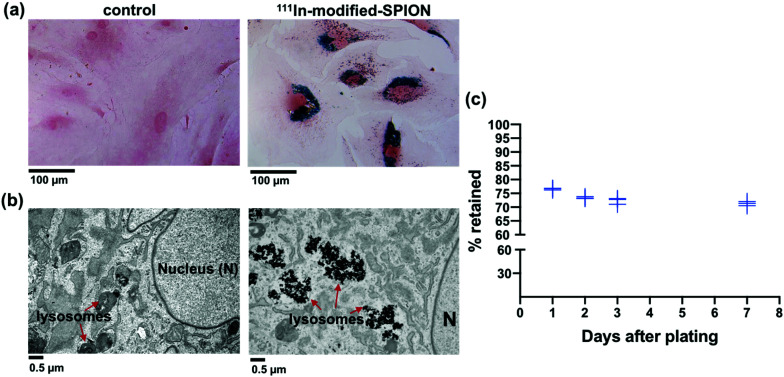
Intracellular uptake and retention of ^111^In-modified-SPION in ADSCs. (a) Prussian blue staining of ADSCs treated with ^111^In-modified-SPION showing blue aggregates compared to untreated control (scale bar = 100 μm). (b) TEM images showing SPION aggregates in lysosomes compared to untreated control (scale bar = 0.5 μm). (c) The percentage of radioactivity retained in ADSCs up to day 7 after labelling with ^111^In-modified-SPION. Data are shown as the replicate values, *n* = 3.

Primary human ADSCs were transduced with a lentiviral vector to express green fluorescence protein (GFP) and firefly luciferase enzyme. The stably transduced ADSCs were incubated with different concentrations of ^111^In-modified-SPION for 16 hours and intracellular iron was measured using the Ferrozine iron assay, which showed a dose-dependent increase in cell uptake (ESI Fig. S5†). The internalisation of ^111^In-modified-SPION by ADSCs was visualised using Prussian blue staining, showing blue aggregates throughout the cytoplasm (Fig. 2a), and TEM, which confirmed that the nanoparticles were contained within lysosomes (Fig. 2b). To study the retention of ^111^In-modified-SPION in ADSCs over time, the media from radiolabelled cells was measured with a gamma counter at days 1, 2, 3 and 7 after plating. The results showed that 73.41 ± 2.16% of radioactivity was retained within ADSCs throughout the study (Fig. 2c).

The effect of ^111^In-modified-SPION labelling on cell migration was evaluated using IBIDI culture inserts and the results showed that there was no significant difference between the control and ^111^In-modified-SPION labelled cells (ESI Fig. S6†). The effect on differentiation was assessed by differentiating control and ^111^In-modified-SPION labelled cells into tri-lineages; adipogenic (oil red O staining), chondrogenic (alcian blue) and osteogenic (alizarin red) (ESI Fig. S7†). Although ^111^In-modified-SPION labelled cells showed a slower rate of chondrogenic differentiation, both the control cells and ^111^In-modified-SPION labelled cells could differentiate towards all three lineages. These findings imply that the labelling process has minimal interference on cell function.

### The effect of radiolabelling on cell viability and cell proliferation

**Fig. 3:**
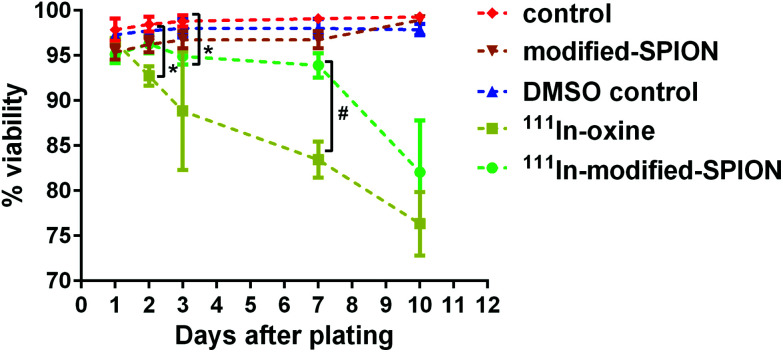
The effect of ^111^In-modified-SPION labelling on cell viability. Live/dead cell viability assay at different time points showed the percentage cell viability of three non-radiolabelled samples: control cells, cells incubated with DMSO (control vehicle for ^111^In-oxine) and modified-SPION labelled cells and two radiolabelled samples: cells labelled with either ^111^In-oxine or ^111^In-modified-SPION (* *P* = 0.0019 ^111^In-oxine *vs.* control at day 2, * *P* = 0.0035 ^111^In-modified-SPION *vs.* control at day 3, # *P* = 0.0016 ^111^In-oxine *vs.*^111^In-modified-SPION at day 7 as measured with multiple *t* test). Data are shown as mean ± SD, *n* = 3.

The major disadvantage of cell tracking with nuclear imaging is cytotoxicity caused by radiolabelling. The cytotoxic effect of ^111^In-oxine in direct stem cell labelling has been demonstrated in many studies.^39^–^41^ In order to compare the level of cytotoxicity between the established radiolabelling method ^111^In-oxine and our method ^111^In-modified-SPION, cell viability and cell proliferation were assessed by using a live/dead cell viability assay and a luciferase assay. The percentage cell viability of two radiolabelled samples (∼10 MBq per million cells for both condition) were compared against three non-radiolabelled samples: cell incubated with DMSO (control vehicle for ^111^In-oxine), modified-SPION labelled cells (to exclude the cytotoxic effect of iron) and control cells (Fig. 3). From day 1 to day 10 after plating, no significant difference in cell viability was observed among three non-radiolabelled samples. In comparison with control cells, ^111^In-oxine labelled cells showed a significant decrease in cell viability from 2 days after plating (*P* = 0.0019). Although ^111^In-modified-SPION labelled cells showed a significant decrease in cell viability from 3 days after plating (*P* = 0.0035), the percentage cell viability is 1.1-fold higher than ^111^In-oxine labelled cells up to 7 days after plating (*P* = 0.0016). Consistent with these findings, the luciferase-based cell proliferation assay also showed the bioluminescence from ^111^In-oxine labelled cells was significantly lower than ^111^In-modified-SPION labelled cells from day 5 onwards (*P* < 0.0001, ESI Fig. S8†). The binding of ^111^In to modified-SPION reduces the cytotoxic effect compared to ^111^In-oxine, allowing for the imaging of viable cells up to 7 days. This is likely to be due to the binding stability of ^111^In-modified-SPION. In ^111^In-oxine chelation, ^111^In dissociates from oxine allowing free ^111^In to bind to cellular components such as DNA leading to rapid cell death.^42^ The cell toxicity is mainly due to low energy Auger electrons emission after radioactive decay.^42^ These emissions are more damaging to DNA when they are present in proximity.^43^

### Assessment of ^111^In-modified-SPION labelled ADSCs distribution following IV or IC injection

**Fig. 4:**
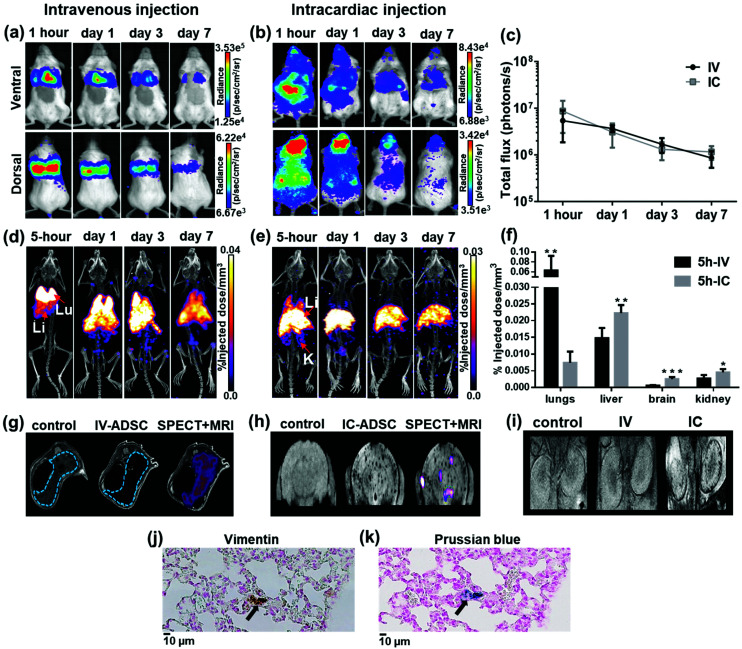
Whole body distribution of ^111^In-modified-SPION labelled ADSCs following IV or IC injection at different time points. (a and b) BLI signal in the chest and the whole body decreased over time. (c) BLI signal from the whole body decreased over time (photons per s in log scale). (d & e) Decay corrected SPECT/CT images of dual labelled ADSCs uptake in organs after IV and IC injection (labels: Lu = lungs, Li = liver, S = spleen and K = kidney). (f) 3D ROI quantification of SPECT signal in lungs, liver, brain and kidney at 5-hour after IV & IC injection calculated as %ID mm^–3^ after decay correction (IV *vs.* IC ** *P* = 0.0027 in lung, ** *P* = 0.0025 in liver, *** *P* = 0.0001 in brain & * *P* = 0.0217 in kidney as measured with unpaired two-tailed *t* test). (g & h) 
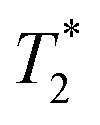
 -weighted MR images of the liver (blue dotted line) & the brain in control and dual labelled cells injected animals with SPECT data co-registered. (i) 
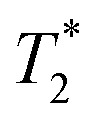
 -weighted MR images of the kidney in a control mouse and mice injected with dual labelled cells intravenously and intracardially. (j & k) Positive vimentin (ADSC) and Prussian blue staining (SPION) of adjacent lung tissue sections showing the presence of dual labelled cells at day 7 after IV injection (indicated by arrows, scale bar = 10 μm). Data are shown as mean ± SD, *n* = 5.

To study *in vivo* distribution of ^111^In-modified-SPION labelled ADSCs, mice were injected with 1 × 10^5^ cells either IV or IC and imaged with BLI, SPECT/CT and MRI serially over 7 days. At 1-hour after IV injection, BLI images showed the majority of cells within the lungs while a wider cell distribution was seen after IC injection (Fig. 4a and b). To assess the effect of labelling agents on cell distribution, BLI results from radiolabelled cells were compared with control ADSCs following IV or IC which showed the similar cell distribution and decreasing signal intensity pattern over 7 days (Fig. 4c & ESI Fig. S9†).

Consistent with data shown by BLI, SPECT signal (%ID mm^–3^) in lungs after 5-hour IV injection was significantly higher than IC injection (*P* = 0.0027, Fig. 4d, e & f) whereas the %ID mm^–3^ in liver, brain and kidney after IC injection was significantly higher than IV injection (*P* = 0.0025, *P* = 0.0001 and *P* = 0.0217 respectively). At days 1, 3 and 7 after IV injection, the %ID mm^–3^ in lung decreased over time, while the signal in liver increased (ESI Fig. S10†). In comparison, the %ID mm^–3^ in liver after IC injection remained stable throughout the study while the signal in lungs, brain and kidney reduced (ESI Fig. S10†). Our findings are in agreement with previous studies on tracking ^111^In-oxine labelled MSCs after IV injection.^9^,^44^ However, most studies have imaged cells in numbers an order of magnitude higher than our study with no known information regarding cell viability. Our study has also identified differences in the ADSCs distribution pattern after IV and IC injection. The main difference being the reduction of ADSC entrapment within the lungs with associated increased retention in the brain and kidney after IC injection. This is a result of administrating the cells through the aorta and the carotid arteries *via* the left ventricle of the heart thereby bypassing the lungs.

Consistent with BLI and SPECT findings, 
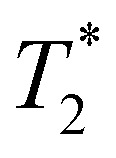
 -weighted MR imaging at day 1 after IV injection showed MR signal loss in liver compared to control but no signal loss was detected in brain and kidney (Fig. 4g, h & i). Conversely, some MR hypointense regions were detected in brain and kidney after IC injection. Distribution within the brain appeared wide-spread, whereas areas of hypointensity were mainly detected within the renal cortex (Fig. 4h and i). To confirm the presence of dual labelled cells, SPECT and 
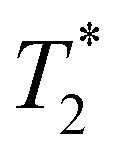
 -weighted MR images of liver and brain were co-registered. The SPECT signal in liver was aligned well with MR signal loss but not all MR hypointense regions in brain were co-registered with SPECT signals (Fig. 4g and h). This demonstrates the difference in sensitivity and the level of information that can be obtained on cell localisation between the two imaging techniques. A focal area of SPION labelled cells can cause sufficient alteration of the MR signal to allow the detection of relatively low numbers of cells, with no corresponding SPECT signal. However, due to MR signal loss and overestimation of the volume due to susceptibility effects, this signal cannot be quantified accurately.^45^

One other discrepancy between imaging modalities was observed. After IV injection, *in vivo* BLI signal was not overlapped with SPECT signal in liver and spleen. This is likely due to BLI signal attenuation by haemoglobin in liver and spleen.^46^ To validate *in vivo* imaging results, *ex vivo* BLI and radioactivity uptake ((injected dose per gram (%ID g^–1^)) in *ex vivo* organs were performed in separate cohorts of mice. The data from *ex vivo* BLI (ESI Fig. S11†) and the %ID g^–1^ from *ex vivo* organs showed patterns of distribution consistent with *in vivo* data (ESI Fig. S10,† full organ data in ESI Tables S1–4†), confirming that viable cells were indeed present in liver and spleen. To confirm the retention of the dual labelling agent within ADSCs, adjacent lung tissue sections at day 7 after IV injection were stained with human specific vimentin (ADSCs) and Prussian blue (SPIONs). Histological images showed areas positive for vimentin staining (Fig. 4j) were also positive for Prussian blue (Fig. 4k). Despite a few discrepancies among imaging modalities, our study has highlighted the advantages of combining imaging techniques to overcome their limitations. By combining direct labelling with reporter gene imaging, cohesive data can be collected on quantitative cell distribution, localisation and cell fate to optimise stem cell-based therapies; to evaluate safety hazards and off-target effects.

### Assessment of ^111^In-modified-SPION labelled ADSCs delivery to tumour following IV or IC injection

Since the IC injection was more efficient than IV in delivering cells to multiple organs, we hypothesised that the IC injection could provide more effective cell delivery to distal tumours. In order to evaluate this, ^111^In-modified-SPION labelled ADSCs (1 × 10^5^ cells) were injected either IV or IC into mice bearing an orthotopic 4T1 breast tumour and imaged with BLI, SPECT and MRI serially over 3 days.

**Fig. 5:**
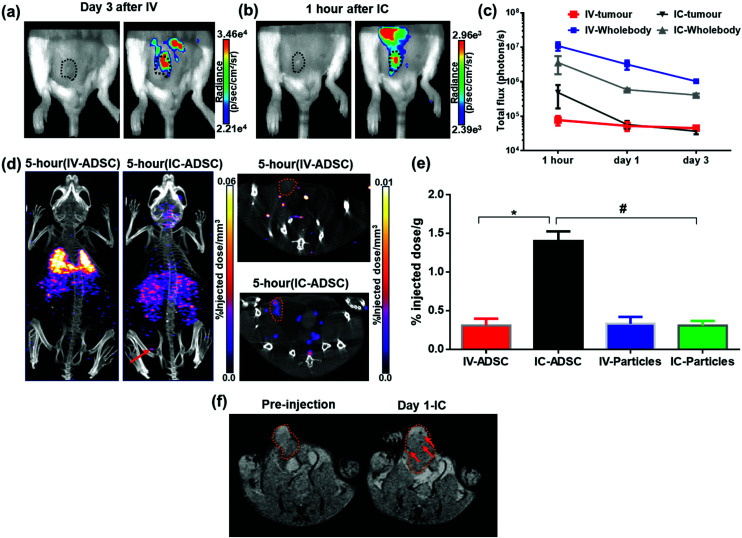
Multi-modal imaging of ^111^In-modified-SPION labelled ADSC distribution in tumour following IV or IC injection. (a & b) BLI signal in tumour at day 3 after IV injection and 1 hour after IC injection (tumour indicated by black dotted lines on the photograph). (c) BLI signal from the tumour and the whole body decreased over time (photons per s in log scale) (d) SPECT/CT images of dual labelled ADSCs at 5-hour after IV and IC injection showing the tumour uptake in a mouse with IC injection (indicated by arrow & orange dotted lines). (e) *Ex vivo* quantification of radioactivity distribution in tumour at 5-hour after IV and IC injection of ^111^In-modified-SPION labelled cells and particles only calculated at %ID g^–1^ after decay correction (* *P* = 0.0002 IV-ADSC *vs.* IC-ADSC, # *P* = 0.0002 IC-ADSC *vs.* IC-Particles as measured with unpaired two-tailed *t* test). (f) 
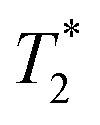
 -weighted MR images of tumour (indicated by orange dotted lines) acquired at pre-injection and day 1 after IC injection of dual labelled ADSCs showing the presence of focal hypointensity within the tumour (indicated by arrows). Data are shown as mean ± SD, *n* = 3.

At 1-hour after IV injection, BLI images showed no signal within the tumour region while high signal was detected after IC injection (Fig. 5a). This process is highly likely to be due to passive retention of cells rather than active migration. Only at day 3 after IV injection, very low signals were detected within tumour area (Fig. 5a). This may suggest migratory capability of ADSCs from lung to tumour. The process of MSC tumour homing is known to be stimulated by inflammatory cytokines and chemokines such as IL6^47^ & SDF-1^48^ released from the tumour followed by the adhesion of circulating MSCs to the vascular wall and transendothelial migration.^49^ However, our imaging data cannot conclusively answer whether this migration process is due to active homing or a passive entrapment mechanism.

BLI signal intensity from tumours and the whole body both decreased at a similar rate overtime (Fig. 5c). The presence of viable cells in tumours at different time points were confirmed by *ex vivo* BLI (ESI Fig. S12†). Although SPECT signal was detected within the tumour at 5-hour after IC injection (Fig. 5d), no signal was able to detect at day 3 after IV injection. Besides, the amount of radioactivity within the tumour was not high enough to perform accurate *in vivo* quantification. 
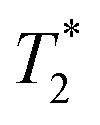
 -weighted MR images of tumours at day 1 after IC injection showed small areas of localised hypointensity throughout the tumour (core as well as rim) compared to the pre-injection image (Fig. 5f). However, no hypointense regions were detected by MRI in the IV injected group at any time point although BLI suggested cell uptake within the tumour at day 3. This is mainly because SPECT and MRI techniques are not as sensitive as BLI in detecting low numbers of cell within tumour.

To eliminate the possibility of free particles being retained by the enhanced permeability and retention (EPR) effect, ^111^In-modified-SPION labelled cells or ^111^In-modified-SPION particles only were injected either IV or IC into tumour bearing mice and *ex vivo* gamma counting (%ID g^–1^) was performed. At 5-hour after IC cell injection, the %ID g^–1^ in tumour was significantly higher than IV cell and IC particle injection (*P* = 0.0002 & *P* = 0.0002, Fig. 5e). No significance difference was observed between the IV cell and IV particle groups. These results are consistent with *in vivo* data and indicate that tumour uptake after IC cell injection is due to cell distribution not free particle accumulation. In addition, vimentin stained tissue sections confirmed the presence of ADSCs within tumour tissue after IC injection (ESI Fig. S12†). Nevertheless, histology staining could not confirm ADSC distribution in the IV injected group presumably because of the low cell numbers. These findings supported our hypothesis that the IC injection route is more efficient than IV in delivering ADSCs to distal tumours.

The arterial route is routinely used to enhance delivery of therapeutic drugs to liver^50^,^51^ and brain tumours^52^,^53^ to exploit the physiological nature of the arterial blood supply. The published literature on MSCs migration to tumours^54^–^56^ has mainly focused on the IV administration route. Using our multi-modal imaging technology, we have shown that IC injection offers a 2-fold higher cell delivery than IV to tumour tissue at the earliest time point (1 hour) when the cells are most viable. The reduction in BLI signal over 7 days was consistent for both injection routes and tumour *versus* non-tumour bearing mice. This suggests that transplanted cells do not remain viable for long periods after injection and this is not dependent on injection route or tumour engraftment. This is in accordance with other studies that have shown the rapid disappearance of MSCs after transplantation.^10^,^57^ This highlights the advantages of combining reporter gene imaging with direct cell labelling for accurately imaging cell fate. Previous studies such as Tang *et al.* have focused on the long-term tracking of MSCs by using a tri-modal probe (SPECT/MRI/fluorescent) incorporating the long half-life radioisotope Iodine-125 but with no correlating information on cell viability.^30^ However, our results and others^10^,^57^ suggest that long-term cell tracking is unnecessary due to the transient viability of transplanted cells.

## Conclusion

In summary, this study highlights how a multi-modal approach can image both location and viability within the same cell population. Detailed information of cell localisation achieved by the combination of two different direct cell labelling and imaging methods (^111^In (SPECT) and SPION (MRI)) was complemented by information on cell viability provided by BLI reporter gene imaging, truly capturing the benefits of a multi-modal imaging approach. Despite the advantages of multi-modal imaging, we have encountered a few limitations such as long scanning time and multiple anaesthesia dose. Due to the differences in sensitivities and labelling methods, inconsistency findings among different imaging modalities can cause misinterpretation of the data. Another challenge in elucidating the imaging data is to exclude the false positive results arising from the release of the direct labelling agent from the dead cells or subsequent uptake by phagocytic cells. Therefore, in this study, a series of validation studies such as histological analysis, *ex vivo* BLI and *ex vivo* gamma counting studies have been performed to confirm *in vivo* imaging results. The major strength of this study was the ability to compare different administration routes to improve stem cell delivery to tumours so that MSC-based cancer therapies can be optimised. Although this study has focused on the use of cell-based tumour therapies, this multi-modal approach can be easily translated to regenerative models and organ systems. Despite some challenges, the advantages of using a multi-modal imaging approach in stem cell tracking would greatly assist the future applications of stem cell-based therapy.

## Experimental

FluidMAG-CT (100 nm, SPION) was obtained from Chemicell, Berlin, Germany. All chemical materials were from Sigma-Aldrich (Dorset, UK), unless otherwise stated and used without further purification.

### Preparation of modified nanoparticles

The synthesis of DOTA-linker is described in ESI-1.† FluidMAG-CT (50 mg mL^–1^; 200 μL) was spun for 5 min and magnetically separated in an Eppendorf tube. H_2_O (500 μL) and MES buffer (100 mM, pH 6, 45 μL) were added. 1-Ethyl-3-(3-dimethylaminopropyl)carbodiimide (4.8 mg) and a solution of *N*-hydroxysulfosuccinimide in H_2_O (230 mM, 17.6 μL) were added and the mixture placed on a shaker bed for 30 min. After this time a strong magnet was used to separate the SPIONs and the reaction solution removed by pipette. The reaction solution was replaced with phosphate buffered saline (PBS, 10 mM, pH 7.4). The nanoparticles were redispersed using a vortexer and separated from the solution using a magnet (repeated twice). Next, the PBS buffer was replaced by DOTA-linker solution in PBS (1 mM, pH 8, 2 mL) and placed on a shaker bed for 3 hours at room temperature. The nanoparticles were magnetically separated by using magnetic separator (MACSiMAG™ Separator – Miltenyi Biotec, Surrey, UK) and washed 3 times with PBS in order to remove unreacted organic molecules and stored in PBS pH 7.4.

### Attempted coupling of Cy3 amine to SPION

Cyanine3 amine was purchased from Lumiprobe. The same procedure was performed as for the modified nanoparticles above using FluidMag CT (25 mg mL^–1^; 200 μL) and Cy3amine (1 mM, 0.7 mg in 1 mL). UV analysis of the Cy3amine stock solution and the solution separated from the nanoparticles after the reaction indicated no change in concentration of Cy3amine and hence that no coupling had occurred.

### Chelation of indium to SPIONs (non-radiolabelled)

The modified SPIONs were incubated for 4 hours with InCl_3_ (1 mM) at 70 °C in 20 mM 4-(2-hydroxyethyl)-1-piperazineethanesulfonic acid (HEPES) buffer (pH 5.5) to ensure maximal indium chelation. Nanoparticles were magnetically separated and washed 3 times with PBS, which was found to be optimal for the removal of free and statically bound In^3+^. In-SPION was synthesised the same way for comparison.

### DLS

The hydrodynamic particle diameter (nm) and the zeta potential (surface charge in mV) of SPIONs only and modified-SPIONs were measured by dynamic light scattering (DLS, Zetasizer Nano-ZS, Malvern Instruments, UK). Both measurements were performed in deionised water at 25 °C in triplicate.

### XPS

X-Ray photoelectron spectroscopy (XPS) was performed using a Thermo Scientific K-alpha spectrometer with monochromated Al Kα radiation, a dual beam charge compensation system and constant pass energy of 50 eV (spot size 400 μm). Samples were mounted on carbon tape. Survey scans were collected in the range 0–1200 eV. High-resolution peaks were used for the principal peaks of In (3d), N (1s). Peaks were modelled with CASA XPS software.

### ICP-AES

All samples were run on a Varian 720 ICP-AES (axial configuration) equipped with autosampler. In-SPIONs and freshly prepared In-modified-SPIONs (8 mg mL^–1^) were suspended in deionised water and aliquots taken at 0, 1, 3 and 7 days to assess the stability of indium chelation. Aliquots (2 mg mL^–1^) were then magnetically separated and washed with water to remove any dissociated indium and resuspended in water. ICP-AES sample prep: the nanoparticles were dissolved in 2% aqua regia (3 : 1 HCl/HNO_3_) and standards prepared containing Fe (0, 4, 8, 12 ppm) and In (0, 1, 2, 3 ppm). From calibration curves the signals at Fe 373.71 and In 303.94 were used for analysis. Samples were run at 100% concentration and 10% concentration and the same In/Fe ratio's obtained.

### *In vitro* assessment of ^111^In-modified-SPION chelation

To assess *in vitro* chelation specificity of modified-SPION to ^111^In, 0.1 mg of modified-SPIONs or SPIONs only were radiolabelled with ∼2 MBq of ^111^InCl_3_ in 20 mM HEPES buffer pH 5.5. After incubation on a shaker for 4 hours at 70 °C, radiolabelling yield of the particles was determined using TLC (Silica gel on TLC Al foils). A pencil line was drawn at 1 cm from the lower end of a TLC strip and 2 μL aliquot of radioactive particles were applied onto it. As a negative control, an equal volume of free ^111^InCl_3_ (without particles) was applied on another strip. When the spot of radioactivity was dry, the tip of the lower end of each TLC strip was submerged in 50 mM diethylenetriaminepentaacetic acid (DTPA) solution (pH 7) to produce the mobile phase. When the solvent almost reached the top of the TLC strip, it was cut at just above the pencil line to get the base (at the point of application) and the top (migrated) part. Then the amount of radioactivity was measured using a gamma counter (Wizard^2^ 3′′, PerkinElmer, Massachusetts, USA) and the percentage of radiolabelling yield was calculated.

### Cell cultures

All experiments were performed using human adipose derived stem cells (ADSCs) which were kindly provided by Dr Michelle Griffin, UCL Plastic and Reconstructive Surgery Department. Lentiviral transduction of ADSCs (passage 2–4) was performed to express green fluorescence protein (GFP) and firefly luciferase enzyme^58^ under the control of the Friend murine leukemia virus FB29 promoter. Using fluorescence microscope (EVOS FL Auto cell imaging system, ThermoFisher Scientific, Massachusetts, USA), the successful transduction was confirmed by GFP expression and the transduction efficiency was 42.56 ± 7.43%. In a humidified incubator at 37 °C with 95% air and 5% CO_2_, the transduced ADSCs (passage 4–7) were grown in T175 flasks (Fisher Scientific, Loughborough, UK) in DMEM-F12, supplemented with 10% fetal calf serum (FCS, Invitrogen, Paisley, UK). Cells were grown to 80% confluence before being trypsinised, centrifuged for pelleting at 300*g*, counted and then plated for *in vitro* studies or re-suspended in PBS for *in vivo* cell injection.

### Lentivirus production

Lentivirus encoding GFP and firefly luciferase was produced in HEK 293T cells using calcium phosphate precipitation protocol adapted from that described by Tiscornia *et al.*,^59^ using the transfer plasmid pSEW-Flagx3-FLuc-2A-GFP (which was a kind gift from Dr Martin Pule, UCL Cancer Institute), together with packaging plasmids, Gag-pol (pCMV-R8.74; Addgene Plasmid # 22036) and VSV-G (pMD2.G; Addgene Plasmid # 12259). Sodium butyrate was added to the media at a final concentration of 1 mM, 24 hours prior to lentiviral harvest, to improve viral titres.^60^ Lentivirus was harvested into ADSC culture medium (DMEM-F12 with 10% FBS), passed through a 20 μm syringe filter, and added directly to ADSCs for transduction. After 24 hours ADSCs were changed into fresh media.

### Ferrozine assay

To measure intracellular iron uptake by ADSCs, the ferrozine assay was performed as previously described.^61^ ADSCs were plated in 24-well plates at a concentration of 0.22 × 10^5^ per well in triplicates and left to attach overnight. The next day, the cells were incubated with ^111^In-modified-SPION either at concentration of 0.1 mg mL^–1^ Fe (∼7 MBq of ^111^In), 0.2 mg mL^–1^ Fe (∼14 MBq of ^111^In) or 0.4 mg mL^–1^ Fe (∼28 MBq of ^111^In) for 16 hours. After washing 3 times with PBS, the labelled ADSCs were trypsinised and pelleted. The cell pellets were frozen and stored at –20 °C. Frozen cells were lysed by adding 50 μL of 1.2 M HCl and incubated for 2 hours at 60 °C. The samples were cooled and centrifuged to collect all condensation. Then 50 μl of deionised water (solvent of SPIONs) was added. To create a standard curve of iron samples with known concentrations, serial dilution of SPIONs at 0, 0.3, 0.625, 1.25, 2.5, 5, 10 and 20 μg mL^–1^ Fe were prepared from the stock solution and 50 μL of 1.2 M HCl was added to each tube. Next, to each tube of cell lysates and SPIONs, 50 μL of iron detection reagent was added and incubated for a further 30 minutes at room temperature. 100 μL of the mixture was then transferred to a 96-well plate and the absorbance was measured at 550 nm and the background at 780 nm using a Varioskan LUX multimode microplate reader (ThermoFisher Scientific, Waltham, MA USA). To calculate the amount of iron in the sample in μg, the background absorbance was subtracted from all samples and a calibration curve for standard was created and liner regression was performed using GraphPad Prism 6. From the equation *y* = *mx* + *c*, where is *m* = slope and *c* = *Y*-intercept, the value of slope was calculated. The absorbance value of the samples was divided by the slope to calculate the amount of iron in μg. Next, the amount of iron in pg per cell (pg Fe per cell) was calculated as follows:
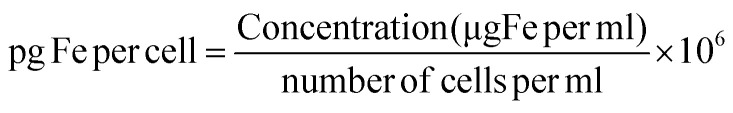


### Perl's Prussian blue staining

To visualise intracellular iron uptake by ADSCs, Prussian blue staining was performed as previously described.^62^ ADSCs were plated in 24-well plates (Corning) at a concentration of 0.22 × 10^5^ per well in triplicates and left to attach overnight. The next day, the cells were incubated with ^111^In-modified-SPION (0.2 mg ml^–1^ Fe, ∼14 MBq of ^111^In) for 16 hours. After washing 3 times with PBS, the labelled ADSCs were fixed in 4% paraformaldehyde (PFA) for 30 minutes at room temperature. The cells were then washed 2 times with PBS followed by staining with Perl's solution for 20 minutes and counterstained with 1% Nuclear Fast Red and then imaged with EVOS FL Auto cell imaging system (ThermoFisher Scientific, Massachusetts, USA).

### Transmission electron microscopy (TEM)

Control ADSCs and ^111^In-modified-SPION labelled ADSCs (∼0.2 MBq of ^111^In, 18 μg of Fe) were seeded at 0.22 × 10^5^ per well on coverslips (VWR) in 24-well plates and then left to attach overnight. Next day the cells were fixed in 2% PFA and 1.5% glutaraldehyde in 0.1 M sodium cacodylate buffer (pH 7.3) for 24 hours at 3 °C then washed 2 times with the same buffer for 30 minutes. The cells were then post fixed in 1% osmium tetraoxide/1.5% potassium ferrocyanide in 0.1 M cacodylate buffer for 1 hour at 3 °C. Then the cells were dehydrated in a graded ethanol-water series and infiltrated with Agar 100 epoxy resin. After dehydration, the cells were placed onto a resin filled Beem capsule with the coverslip side down and hardened at 60 °C for 48 hours and then the coverslips were removed by hydrochloric acid (HCl). A representative area was selected and ultra-thin sections were cut at 70–80 nm using a diamond knife on a Reichert ultra-cut S microtome. Sections were collected on 300 mesh copper and stained with lead citrate. Then viewed with a Joel 1010 transition electron microscope and the images were recorded using a Gatan Orius CCD camera.

### Migration assay

To assess the migration capability of ADSCs after dual labelling, ^111^In-modified-SPION labelled ADSCs (∼0.1 MBq of ^111^In, 9 μg of Fe per well) were seeded at a concentration of 0.1 × 10^5^ per well in 4-well silicone inserts with 4 defined 500 μm cell-free gaps (IBIDI, Martinsried, Germany). The inserts were removed the next day and cell migration was monitored. After 30 hours, the cells were stained with Hoechst 33342 (ThermoFisher Scientific, Massachusetts, USA) and the number of cells between the gaps was analysed using Image J software and compared with the results from control ADSCs.

### Differentiation assay

To determine the effect of labelling on the differentiation potential of ADSCs, a differentiation assay was performed as previously described.^63^ ADSCs were labelled with ^111^In-modified-SPION (∼0.2 MBq of ^111^In and 18 μg of Fe) and plated in 24-well plates (Corning) at a concentration of 0.22 × 10^5^ per well in triplicates. When both labelled and control cells reached 90–100% confluency, the regular media was removed and differentiation media (adipogenic, chondrogenic or osteogenic) was added to appropriate wells and was changed every three days. Undifferentiated wells received regular media. After 3 weeks of changing media, staining was conducted for each differentiation, the cells were imaged using the EVOS FL Auto cell imaging system.

### Adipogenic differentiation

Adipogenic differentiation was induced with DMEM-F12 medium containing 10% FCS, 1% penicillin/streptomycin, 10 ng mL^–1^ insulin, 1 μM dexamethasone, 500 μM 3-isobutyl-1-methylxanthine and 1 mM rosiglitazone. After 3 weeks, cells were fixed in 10% formalin for 30 minutes, washed with deionised water and then washed again with 60% isopropanol for 5 minutes prior to staining with Oil Red O working solution for 10 minutes. After staining, cells were washed several times with tap water.

### Chondrogenic differentiation

Chondrogenic differentiation was induced with DMEM-F12 medium containing 10% FCS, 1% penicillin/streptomycin, 0.1 μM dexamethasone, 50 μg mL^–1^ ascorbate, 10 ng mL^–1^ transforming growth factor (TGF) β1 (Life technologies, Paisley, UK) and insulin, transferrin, selenium. After 3 weeks, cells were fixed in 4% paraformaldehyde (PFA) for 30 minutes and then washed with deionised water. Then rinsed with 0.1 M HCl for 5 minutes and stained with Alcian Blue staining (1% in 0.1 M HCl) for 30 minutes. After staining the cells were washed with tap water 3 times.

### Osteogenic differentiation

Osteogenic differentiation was induced with DMEM-F12 medium containing 10% FCS, 1% penicillin/streptomycin, 0.1 μM dexamethasone, 10 mM β-glycerophosphate and 100 μg mL^–1^ ascorbate. After 3 weeks, cells were fixed in ice-cold 70% ethanol for 1 hour and washed with deionised water. Then the cells were stained with Alizarin Red staining (1% in deionised water, pH 4.1–4.3). After staining, cells were washed with tap water 3 times.

### Retention of ^111^In-modified-SPIONs after intracellular uptake

To study the leakage of radioactivity from ^111^In-modified-SPION after intracellular uptake, the radiolabelled cells were plated in triplicates as described above. The media was removed and measured with a gamma counter at days 1, 2, 3 and 7 after plating. The percentage of radioactivity retained was calculated by subtracting the amount of radioactivity in the media from the initial activity measure in the labelled cells at day 0 after decay correction and multiplied by 100.

### ADSC labelling with ^111^In-oxine

50.16 MBq of ^111^InCl_3_ in 1 M HCl was diluted to 500 μL with HPLC grade water, and neutralised using 1 μL aliquots of 1 M NaOH. Next, 5 μL of a 10 mg ml^–1^ solution of 8-hydroxyquinoline in chloroform was added and vortexed in a round-bottomed Pyrex tube for 5 minutes. 500 μL of chloroform was added followed by a brief centrifugation at 300*g* and vortexing for 25 minutes. The chloroform phase containing the ^111^In-oxine complex was extracted from below the water phase containing free ^111^In, and evaporated at 80 °C in a conical bottom HPLC vial, before resuspension in 20 μL of dimethyl sulfoxide (DMSO). ADSCs were trypsinised, counted, re-suspended in PBS and incubated with desired amount of ^111^In-oxine at 37 °C for 30 minutes. Subsequently, the cells were pelleted and washed 3 times with PBS and the radioactivity of the cell pellets was measured using an isotope calibrator (Curiementor 4, PTW). The labelling yield was ∼10 MBq per million cells.

### *In vitro* cell viability assay

For cell viability assay, ADSCs (5 × 10^3^ cells per well) were labelled with either ^111^In-oxine (∼0.05 MBq) or ^111^In-modified-SPION (∼0.05 MBq of ^111^In, ∼4.5 μg of Fe) and were plated at the same time with three non-radiolabelled samples: control cells, cells incubated with 1 : 200 of DMSO (control vehicle for ^111^In-oxine) and modified-SPION labelled cells (∼4.5 μg of Fe) in 96-well plates (Corning) in triplicates. Cell viability assay was performed using Propidium Iodide (Biotium, Fremont, USA) and Hoechst 33342 (NucBlue™ live cell stain, ThermoFisher Scientific, Massachusetts, USA) at days 1, 2, 3, 7 and 10 after plating. To stain the dead cells, the cells were incubated with 5 μM Propidium Iodide (150 μl per well) for 10 minutes followed by live cell staining using Hoechst 33342 (20 μL per well) which was incubated for 20 minutes. The cells were imaged using EVOS FL cell imaging system (ThermoFisher Scientific, Massachusetts, USA) and images were analysed using Image J software (version 1.46r) (ImageJ, U.S. National Institutes of Health, Bethesda, Maryland, USA. The results were calculated as below and presented as the percentage cell viability.
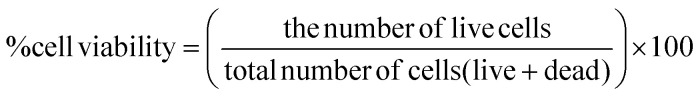


### *In vitro* luciferase assay

For luciferase-based cell proliferation assay, ADSCs (0.5 × 10^5^ cells per well) were labelled with ^111^In-oxine (∼0.5 MBq), ^111^In-modified-SPION (∼0.5 MBq of ^111^In, ∼45 μg of Fe) and three non-radiolabelled samples: control cells, cells incubated with 20 μL of DMSO (control vehicle for ^111^In-oxine) and modified-SPION labelled cells (∼45 μg of Fe) were plated in 24-well plates (Corning) in triplicates. *In vitro* luciferase assay was performed using AMI-X (Spectral Instruments Imaging, USA) at days 1, 3, 5, 6, 7, 8 and 10. Images were acquired immediately after adding d-luciferin (60 μg mL^–1^, Beetle Luciferin Potassium Salt, Promega, Madison, WI) using medium or low binning and exposure time of 120 s. A region of interest (ROI) was placed over each well and the total signal in the ROI (photons per s) was quantified using AMIView software (version 1.7.07).

### *In vivo* studies

All the animal procedures were approved by local Animal Welfare and Ethical Review Bodies at University College London and licensed under the UK Home Office regulations and the Guidance for the Operation of Animals (Scientific Procedures) Act 1986 and following Guidelines for the Welfare and Use of Animals in Cancer Research. All mice were 6–8-week-old female non-obese diabetic/severe combined immunodeficiency/gamma (NSG) mice (Charles River Laboratories, UK). All *in vivo* imaging experiments were performed under isoflurane anaesthesia (1.5%–2.5% isoflurane in oxygen 1.5–2 L min^–1^).

### Assessment of ^111^In-modified-SPION chelation stability in *in vivo*

To assess *in vivo* chelation stability of modified-SPION to ^111^In, mice were injected intravenously *via* tail vein (150 μL PBS) with either ∼2.5 MBq of ^111^InCl_3_ (*n* = 3) or ∼2 MBq ^111^In-modified-SPION particles containing ∼142.8 μg of Fe (*n* = 3).

### ADSCs labelling with ^111^In-modified-SPIONs for *in vivo* experiment

For ADSCs labelling, 5 mg of modified-SPIONs were used for radiolabeling with ∼70 MBq of ^111^InCl_3_ as described above and the radiolabelled particles were washed 3 times with PBS to remove free ^111^InCl_3_. ∼60 MBq of ^111^In-modified-SPIONs (∼0.33 mg ml^–1^ of Fe in 15 ml of media) was incubated with ADSCs (7.5 × 10^5^ cells) at 37 °C for 16 hours. Labelled ADSCs were washed 3 times with PBS to remove excess particles and trypsinised, pelleted and washed again 3 times with PBS. The radioactivity of the final cell pellets was measured prior to further investigations. The labelling yield was ∼10 MBq per million cells.

### *In vivo* cell distribution studies in healthy animals

For *in vivo* distribution studies of non-radiolabelled ADSCs, mice were injected with 1 × 10^5^ cells in 100 μL of PBS either intravenously (*n* = 4) *via* a tail vein or intracardially (*n* = 3) through the left ventricular cavity of the heart with ultrasound guidance (VEVO 2100; Visualsonics, Canada) and imaged with BLI for 7 days.

For *in vivo* distribution studies of ^111^In-modified-SPION labelled ADSCs, mice were injected with 1 × 10^5^ dual labelled cells either intravenously (∼1.34 MBq of ^111^In, ∼140 μg of Fe, *n* = 5) or intracardially (∼0.83 MBq of ^111^In, ∼100 μg of Fe, *n* = 5). The animals were imaged with BLI, SPECT/CT and MRI serially over 7 days.

### *In vivo* cell distribution studies in tumour bearing animals

To develop an orthotopic primary breast tumour model, 4T1 murine breast cancer cells (a kind gift from Dr Elnaz Yaghini, UCL Surgery and Interventional Science) were grown in T75 flasks (Fisher Scientific, Loughborough, UK) in Dulbecco's modified Eagles medium (DMEM) (Invitrogen, Paisley, UK), supplemented with 10% fetal calf serum (FCS, Invitrogen, Paisley, UK) in a humidified incubator at 37 °C with 95% air and 5% CO_2_. Cells were grown to 80% confluence before being trypsinised, centrifuged for pelleting at 300*g*, counted and then re-suspended in PBS at a concentration of 0.5 × 10^5^ cells/50 μL. Animals were anesthetised and the area around the 4^th^ and 5^th^ mammary gland was shaved and the cell suspension was injected into right 5^th^ mammary fat pads. Tumour growth was monitored using MRI at days 9 and 12 after tumour implantation. The average tumour size used in the studies was ∼40 mm^3^ and tumour sizes were matched between IV and IC injected groups.

For *in vivo* cell distribution studies of ^111^In-modified-SPION labelled ADSCs, the tumour-bearing mice were injected either IV (*n* = 3) or IC (*n* = 3) with 1 × 10^5^ dual labelled cells (∼0.5 MBq of ^111^In, 52 μg of Fe). The mice were imaged with BLI, SPECT/CT and MRI serially over 3 days. The imaging sessions were terminated at 3 days after cell infusion as in the pilot studies some NSG mice became weak and were unable to tolerate anesthesia at 14 days after tumour implantation.

### Longitudinal *in vivo* imaging time points

All *in vivo* BLI and SPECT/CT imaging were performed at 1-hour, days 1, 3 and 7 after injection in studies with healthy animals and at 1 hour, days 1 and 3 after injection in studies with tumour bearing animals except the earliest time point for SPECT/CT imaging was at 5-hour after injection. All MRI was performed at days 1 and 3 after injection.

### BLI

Mice were anaesthetised and *in vivo* BLI was performed using IVIS Lumina (PerkinElmer, USA). Mice were injected intraperitoneally with 75 mg kg^–1^d-luciferin (Promega) in 200 μL of PBS. Sequential BLI images were acquired 5 minutes after luciferin injection using auto exposure time with 0.5 minutes delay between two consecutive acquisitions. A rectangular region of interest (ROI) was placed over the whole body on the first image and subsequently pasted over every new image acquired until all ROIs reach their maximum intensity. In tumour studies, a circular ROI was placed over the tumour. The total signal in the ROI was quantified as total flux (photons per s) by using Living Image software version 4.5 (PerkinElmer). Representative images were presented using radiance (p s^–1^ cm^–2^ sr^–1^) as color scale by utilising the same software.

### SPECT/CT

Mice were anaesthetised and whole-body SPECT/CT scans were acquired using a NanoSPECT/CT scanner (Mediso, Hungary). CT images were acquired using a 45 kilo volt peak (kVp) X-ray source, 500 ms exposure time in 180 projections, a pitch of 1.5 with an acquisition time of 7 minutes. SPECT images were obtained with a 4-head scanner with nine 1.4 mm pinhole apertures in helical scan mode using a time per view of 60–90 seconds resulting in a scan time of 36–60 minutes and respiration was monitored throughout the scan and body temperature maintained with a warm air blower. CT images were reconstructed in voxel size 124 × 124 × 124 μm using Bioscan InVivoScope (Bioscan, USA) software, whereas SPECT images were reconstructed in a 256 × 256 matrix using HiSPECT (ScivisGmbH, Bioscan). Images were fused and analysed using InVivoScope (Version 1.44, Bioscan). 3D ROIs were created for brain, lungs, liver and kidney for each time point using VivoQuant (inviCRO version 2.5 patch 1) software. After correcting for ^111^In decay, the 3D ROIs were calculated as the percentage of injected dose per mm^3^ (%ID mm^–3^). Representative maximum intensity projection (MIP) images were presented using %ID mm^–3^ as color scale after decay correction and Guassian filtering of 0.8 by utilising the same software.

### MRI

Mice were anaesthetised and positioned in a custom cradle with temperature and respiration monitoring. MRI studies were performed using a 9.4T Varian scanner (Agilent, USA) for liver and brain imaging and a 1T ICON scanner (Bruker, Germany) for kidney and tumour imaging. For liver imaging, a 35 mm volume radiofrequency coil (Rapid, Germany) and respiration-gated sequences were used. 
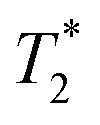
 -weighted liver images were acquired using a fast spin echo multi slice sequence with the following parameters: repetition time (TR) = 1000 ms; echo time (TE) = 20 ms; spatial resolution = 115 μm per pixel; slice thickness = 1 mm. For brain imaging, a 72 mm volume radiofrequency coil was used with a surface receive coil positioned over the skull. 
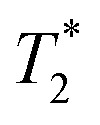
 -weighted brain images were acquired with a gradient echo sequence using TR = 500 ms; TE = 4.1 ms; in plane resolution = 130 μm^2^; slice thickness = 0.6 mm. 
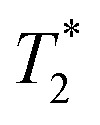
 -weighted kidney and tumour images were acquired using TR = 147.5 ms; TE = 5 ms; spatial resolution = 216 μm per pixel; slice thickness = 1 mm. For monitoring tumour growth, *T*_2_-rapid acquisitions with relaxation enhancement (RARE) images of tumour were acquired at days 9 and 12 after tumour implantation using the following parameters: TR = 2483 ms; TE = 16.5 ms; rare factor = 8; spatial resolution = 287 μm per pixel; slice thickness = 1 mm. Acquisition time = ∼15 minutes for each scan. The volumetric measurement of the tumour was calculated using ImageJ software.

### *Ex vivo* BLI and *ex vivo* biodistribution study

All *ex vivo* BLI and *ex vivo* biodistribution studies were performed at 1 hour, days 1, 3 and 7 after injection in studies with healthy animals and at 1 hour and day 3 after injection in studies with tumour bearing animals (*n* = 3 for each time point in both studies). Under anesthesia, mice were injected intraperitoneally with 75 mg kg^–1^d-luciferin. At 10 minutes after injection, mice were sacrificed and the organs of interest were excised quickly and *ex vivo* BLI was performed using 5 minutes exposure time and binning 8. Directly after *ex vivo* BLI, each sample was weighed and counted with a gamma counter and the amount of radioactivity was decay corrected and calculated as the percentage of injected dose per gram (%ID g^–1^).

### Histological analysis

At the end of imaging sessions, selected mice were sacrificed and tissue samples were excised, fixed in 10% neutral buffered formalin for 24 hours and embedded in paraffin. Then 5 μm tissue sections were prepared for different histological staining. To assess morphological changes, haematoxylin and eosin (H&E) staining was performed. Human specific vimentin (CONFIRM™ anti-Vimentin (V9) primary mouse monoclonal antibody, Roche) was performed to detect the presence of ADSCs in tissue sections using Ventana Discovery XT instrument and the Ventana DAB Map detection Kit (for lung sections) or Ventana Red detection kit (for tumour sections). For pre-treatment, Ventana CC1, equivalent to EDTA buffer, was used. The slides were counterstained with hematoxylin. The adjacent 5 μm sections from immunohistochemistry (IHC) stained sections were stained with Prussian blue staining to demonstrate the presence of SPIONs. In brief, deparaffinised and hydrated sections were immersed in the freshly prepared Perl's solution (equal part of 2% hydrochloric acid and 2% potassium ferrocyanide) for 10 minutes and washed 3 times with distilled water. Next the slides were counterstained with nuclear fast red for 1 minute and washed 2 times with distilled water. Then the slides were mounted with resinous mounting medium and scanned with Nanozoomer slide scanner (Hamamatsu Photonics, Japan). The images were viewed with NanoZoomer Digital Pathology software (NDP Version 2.7.25).

### Statistical analysis

All *in vitro* experiments were repeated at least 3 times with 3 triplicates.

Statistical analysis was performed with GraphPad Prism version 6.01. Data were presented as mean ± standard deviation (SD). Multiple *t* test was conducted for cell death and cell proliferation assays and unpaired two-tailed *t* test was conducted in the rest of the experiments.

## Author contributions

M. Z. T. and T. L. K. conceived the experiments and wrote the manuscript, H. A., R. B., T. D. K., and S. G. performed the synthesis and characterisation of the particles, M. Z. T., J. J. C., and D. J. S. conducted the experiments and edited the paper, P. S. P. performed lentiviral transduction and edited the paper, H. C. H., A. B. T., and M. F. L. supervised the study and assisted in writing manuscript.

## Conflicts of interest

The authors declare no competing financial interest.

## Supplementary Material

Supplementary information
