# Supplementary material for: Multi-modal imaging probe for assessing the efficiency of stem cell delivery to orthotopic breast tumours
Source: Nanoscale. 2020 Aug 4;12(31):16570–85. doi: 10.1039/d0nr03237a (PMC7586303; doi:10.1039/d0nr03237a)
Supplement: Supplementary file 1 [file NR-012-D0NR03237A-s001.pdf]

Electronic Supplementary Material (ESI) for Nanoscale.
This journal is © The Royal Society of Chemistry 2020

Multi-modal Imaging Probe for Assessing the Efficiency of Stem Cell Delivery to Orthotopic Breast Tumours

Supplementary Data

May Zaw Thin[1*], Helen Allan[2], Robin Bofinger[2], Tomas D. Kostelec[2], Simon Guillaume[2], John J. Connell[1], P. Stephen Patrick[1], Helen C. Hailes[2], Alethea B. Tabor[2], Mark F. Lythgoe[1], Daniel J. Stuckey[1] and Tammy L. Kalber[1*]

[1] UCL Centre for Advanced Biomedical Imaging, Division of Medicine, University College London, London, WC1E 6DD, UK,

[2] Department of Chemistry, University College London, 20, Gordon Street, London, WC1H 0AJ, UK,

Corresponding author: t.kalber@ucl.ac.uk; may.zawthin@crick.ac.uk

Keywords: cell tracking, multi-modal imaging, stem cell therapy, nanoparticles, reporter gene imaging

Supplementary Information – 1

Preparation of DOTA-linker

We have previously described[1] the preparation of liposome-DOTA conjugates for multimodal imaging, in which the DOTA is attached to the surface of the liposome by a short n-ethylene glycol (n-EG) spacer of defined length. This gives shielded nanoparticles with a shallow but even coverage of n-EG, which showed good cellular internalization in a range of tumor cells. Here, we have adapted this approach to prepare DOTA-SPION bioconjugates.

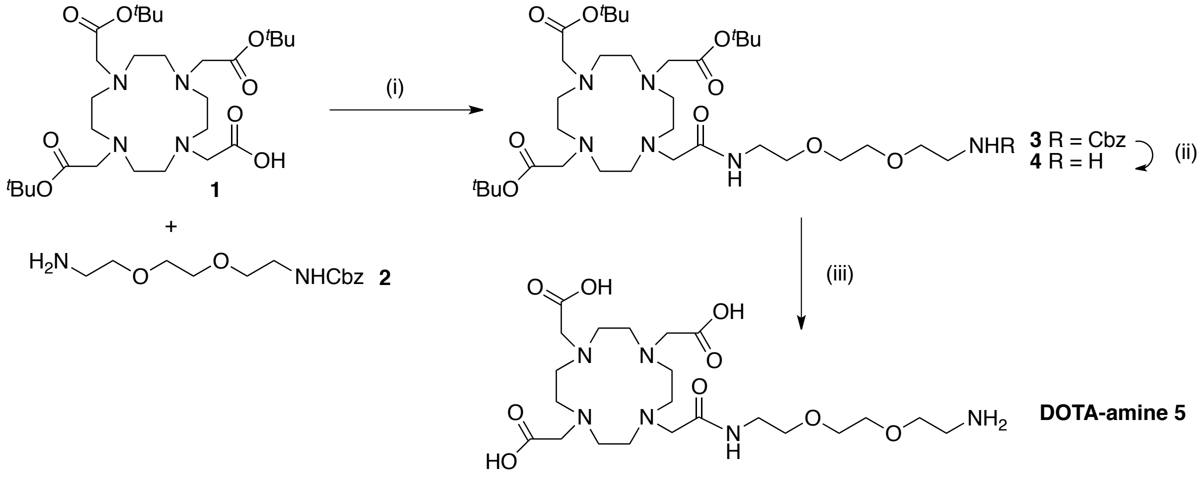

Scheme 1. Synthesis of DOTA-amine **5**. (i) DIC, NHS, DIPEA, CH_2Cl_2 (61%); (ii) H_2, Pd/C, MeOH (48%); (iii) TFA, CH_2Cl_2 (quantitative).

General Experimental

Reagents for chemical synthesis were purchased from Sigma-Aldrich Co. Ltd. unless otherwise stated and used without further purification. All reagents were of commercial quality and used as received and all solvents anhydrous. Thin Layer Chromatography (TLC) was performed on aluminium backed Sigma-Aldrich TLC plates with F254 fluorescent indicator. Visualisation was performed by quenching of UV fluorescence or by staining the plates with potassium permanganate solution (1.5 g $KMnO_4$, 10 g K_2CO_3, 1.25 mL 10% NaOH in 200 mL water), phosphomolybdic acid solution (10% w/w in ethanol). Normal phase flash chromatography was

carried out using silica gel (43–60 µm) supplied by Merck. Infrared spectra were recorded using a Bruker Alpha ATR spectrometer. ^{1}H and ^{13}C NMR spectra were recorded on a Bruker Avance III 600 or 400 instrument, as stated, at the field indicated. Chemical shifts (in ppm) were referenced to residual protonated solvent. Coupling constants (*J*) were measured in Hertz (Hz), multiplicities for ^{1}H coupling are shown as s (singlet), d (doublet), t (triplet), m (multiplet), or a combination of the above. Deuterated chloroform ($CDCl_3$) was used as a solvent. ESI-MS analysis was performed on a Agilent 6510 QTOF mass spectrometer. EI-MS analysis was performed on a Thermo Finingan MAT900 magnetic sector mass spectrometer.

Synthesis of DOTA-amine 5

Tri-*tert*-butyl 2,2',2''-(10-(3,14-dioxo-1-phenyl-2,7,10-trioxa-4,13-diazapentadecan-15-yl)-1,4,7,10-tetraazacyclododecane-1,4,7-triyl)triacetate (**3**)

A solution of **1**[1] (1.2 g, 2.1 mmol), **2**[2] (0.65 g, 0.23 mmol), HBTU (1.2 g , 3.2 mmol), and TEA (0.61 ml, 0.47 g, 4.2 mmol) in CH_2Cl_2 (30 mL) and DMF (3 mL) was left stirring overnight at room temperature. The reaction mixture was then diluted with DCM (50 mL) and washed with brine (3 × 15 mL). The organic layer was dried ($MgSO_4$), filtered and the solvent removed under reduced pressure. The crude was purified via silica column chromatography (R_f = 0.48 in 8% MeOH/CH_2Cl_2; column solvent: gradient 5-8% MeOH/CH_2Cl_2) and **3** was collected as a pale yellow oil (117 mg, 0.140 mmol, 61%).

IR v_{max} 3420 (N-H stretch), 3328 (N-H stretch), 2820-2980 (C-H), 1723 (C=O), 1675 (C=O), 1531 (N-H bend), 1306 (C-N), 1227 (C-O), 1157 (C-O); ^{1}H NMR (400 MHz, 60 ºC, $CDCl_3$) δ 7.42-7.29 (5H, m, ArC*H*), 5.23 (1H, s, NH), 5.14 (2H, s, COOCH_2Ph), 3.68-3.53 (8H, m, CH_2OC_2H_4OCH_2), 3.44-3.48 (4H, m, NHCH_2CH$_2$O, OCH$_2$CH_2NH$_2$), 3.27-2.97 (8H, br, NCH_2COOtBu, NCH_2CON), 2.79-2.20 (16H, br, NC_2H_4N), 1.50 (9H, s, C(CH_3)$_3$), 1.48 (18H, s, C(CH_3)$_3$); ^{13}C NMR (150.9 MHz, $CDCl_3$) δ 172.6 (NCH$_2$*C*OOtBu), 171.9 (NCH$_2$*C*ONH), 156.6 (Ar*C*), 136.7 (ipso-Ar*C*), 128.6 (Ar*C*), 128.1 (Ar*C*), 81.9 (*C*(CH$_3$)$_3$), 70.3 (OCH$_2$CH$_2$O), 70.3 (OCH$_2$*C*H$_2$O), 70.0 (HNCH$_2$*C*H$_2$O), 69.4

(HNCH$_2$*C*H$_2$O), 66.7 (COO*C*H$_2$Ph), 48.0-57.0 (DOTA, br), 40.9 (NH*C*H$_2$CH$_2$O), 39.2 (OCH$_2$*C*H$_2$NH), 28.1 (*C*H$_3$), 28.0 (*C*H$_3$); m/z (ESI+ HRMS) found 837.5363 (100%, $[M+H]^+$), $C_{42}H_{72}N_6O_{11}$ requires 837.5337.

Tri-*tert*-butyl 2,2',2''-(10-(2-((2-(2-(2-aminoethoxy)ethoxy)ethyl)amino)-2-oxoethyl)-1,4,7,10-tetraazacyclododecane-1,4,7-triyl)triacetate (**4**)[3,4]

Compound **3** (0.092 g, 0.11 mmol) was dissolved in MeOH (5 mL) in a round bottom flask and Pd/C (0.01 g) was added. The flask was sealed with a suba-seal. The atmosphere in the flask was replaced with hydrogen by evacuating the flask and purging it with hydrogen (3 times). The reaction was left vigorously stirring for 4.5 h under a hydrogen atmosphere. The mixture was filtered through Celite and the solvent in the filtrate removed under reduced pressure. The crude transparent oil was purified via silica column chromatography (20% MeOH/CH_2Cl_2; R_f = 0, then DCM: EtOH: NH_3 (aq) – 1.0: 0.7: 0.2; R_f = 0.2). The product **4** was collected as a colourless oil (0.037 g, 0.053 mmol, 48%).

IR v_{max} 3393 (N-H stretch), 3236 (N-H stretch), 2800-3050 (C-H), 1727 (C=O), 1671 (C=O), 1225 (C-N), 1155 (C-O) cm^{-1}; ^{1}H NMR (400 MHz, 60 ºC, $CDCl_3$) δ 3.72-3.58 (8H, m, C*H$_2$*OC$_2$*H$_4$*OC*H$_2$*), 3.58-2.27 (28H, m, DOTA, CONHC*H$_2$*, OCH$_2$C*H$_2$*NH$_2$), 1.50 (9H, s, C(C*H$_3$*)$_3$), 1.49 (18H, s, C(C*H$_3$*)$_3$); ^{13}C NMR (150.9 MHz, $CDCl_3$) δ 172.6 (NCH$_2$*C*OOtBu), 172.2 (NCH$_2$*C*ONH), 81.9 (*C*(CH$_3$)$_3$), 81.9 (*C*(CH$_3$)$_3$), 70.1 (*C*H$_2$O), 70.0 (*C*H$_2$O), 66.3 (*C*H$_2$O), 48.0-57.0 (DOTA, br), 40.9 (NH*C*H$_2$CH$_2$O), 39.0 (OCH$_2$*C*H$_2$NH), 28.1 (*C*H$_3$); m/z (ESI+ HRMS) 703.4958 (100%, $[M+H]^+$), $C_{34}H_{66}N_6O_9$ requires 703.4970;

2,2',2''-(10-(2-((2-(2-(2-aminoethoxy)ethoxy)ethyl)amino)-2-oxoethyl)-1,4,7,10-tetraazacyclododecane-1,4,7-triyl)triacetate (**5**)

The *tert*-butyl protected compound **4** (260 mg, 0.37 mmol) was dissolved in a 1:1 mixture of DCM and TFA (1 mL) The reaction mixture was stirred at room temperature overnight. The solvent was then evaporated and the residue redissolved in chloroform. After the solvent had evaporated the title compound **5** was isolated as an amorphous solid (240 mg, 0.37 mmol, quant.).

IR ν_{max} 3550-3000 (br, O-H, N-H stretch), 2870 (C-H), 1722 (C=O), 1668 (C=O), 1458, 1289, 1197, 1130, 1086; ^{1}H NMR (700 MHz, 60 ºC, MeOD) δ 4.05-3.75 (8H, br, DOTA), 3.78-3.75 (2H, m, $OCH_2CH_2NH_3$), 3.73-3.67 (4H, m, OCH_2CH_2O), 3.62 (2H, t, *J* = 5.6, OCH_2CH_2NH), 3.45 (2H, t, *J* = 5.6, OCH_2CH_2NH), 3.50-3.15 (16H, br, DOTA), 3.19-3.17 (2H, m, $OCH_2CH_2NH_3$); ^{13}C NMR (175 MHz, 60 ºC, MeOD) δ 171.7 (br, $NCH_2COOtBu$, NCH_2CONH), 162.3 (q, *C*(O)CF_3), 117.6 (q, C(O)CF_3), 71.4 (OCH_2CH_2O), 71.3 (OCH_2CH_2O), 70.5 (OCH_2CH_2NHCO), 67.7 ($OCH_2CH_2NH_3^+$), 56.1(NCH_2CONH), 55.0-55.5 (br, DOTA), 51.5 (br, DOTA), 51.2 (br, DOTA), 51.0 (br, DOTA), 40.6 (OCH_2CH_2NHCO), 40.3 ($OCH_2CH_2NH_3^+$); m/z (ES- HRMS) found 533.2933 (100%, $[M-H]^+$), $C_{22}H_{41}N_6O_9$ requires 533.2935.

NMR Spectra

^{1}H NMR: Tri-*tert*-butyl 2,2',2''-(10-(3,14-dioxo-1-phenyl-2,7,10-trioxa-4,13-diazapentadecan-15-yl)-1,4,7,10-tetraazacyclododecane-1,4,7-triyl)triacetate at 60 °C (**3**)

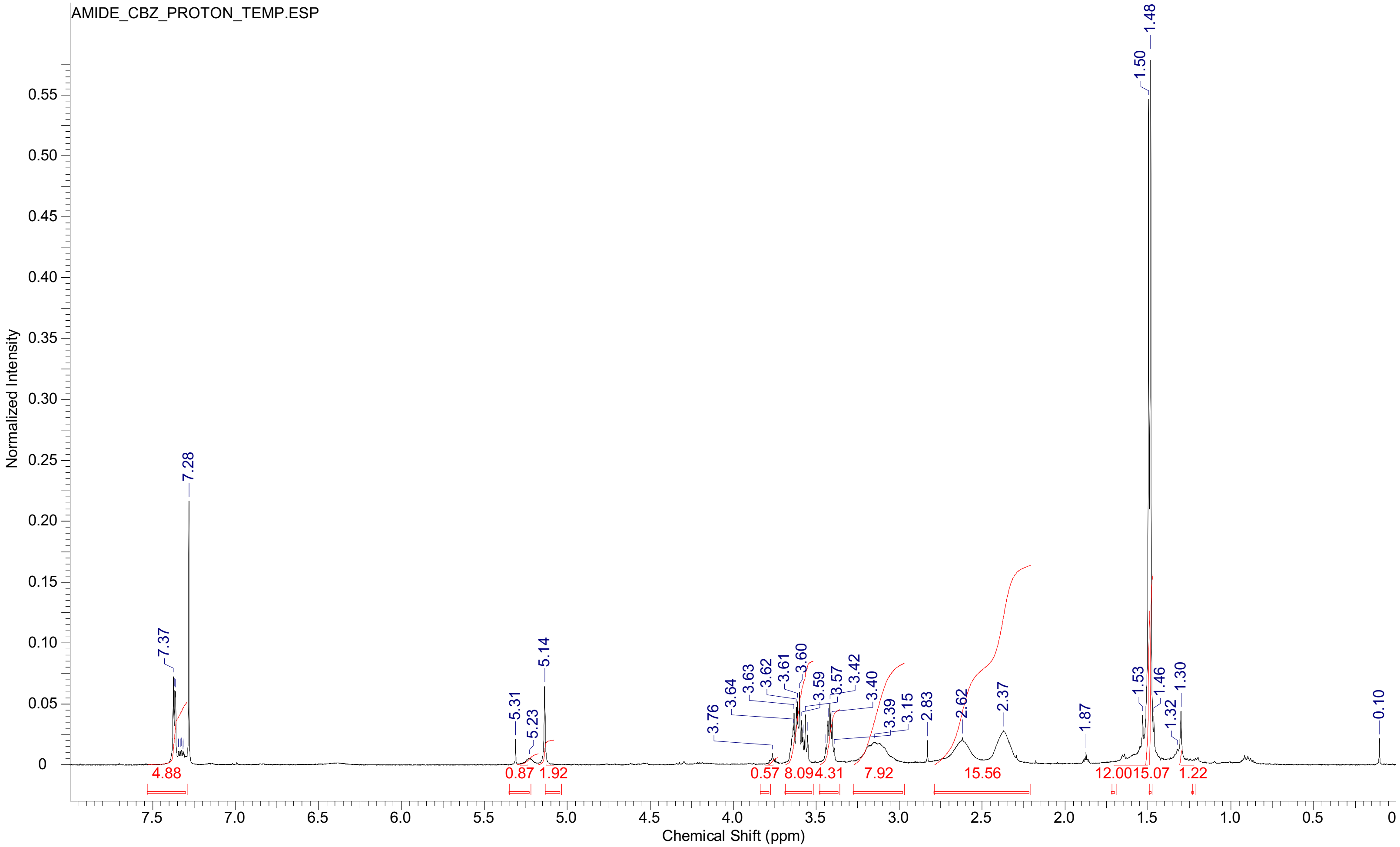

^{1}H NMR: Tri-*tert*-butyl 2,2',2''-(10-(3,14-dioxo-1-phenyl-2,7,10-trioxa-4,13-diazapentadecan-15-yl)-1,4,7,10-tetraazacyclododecane-1,4,7-triyl)triacetate (**3**)

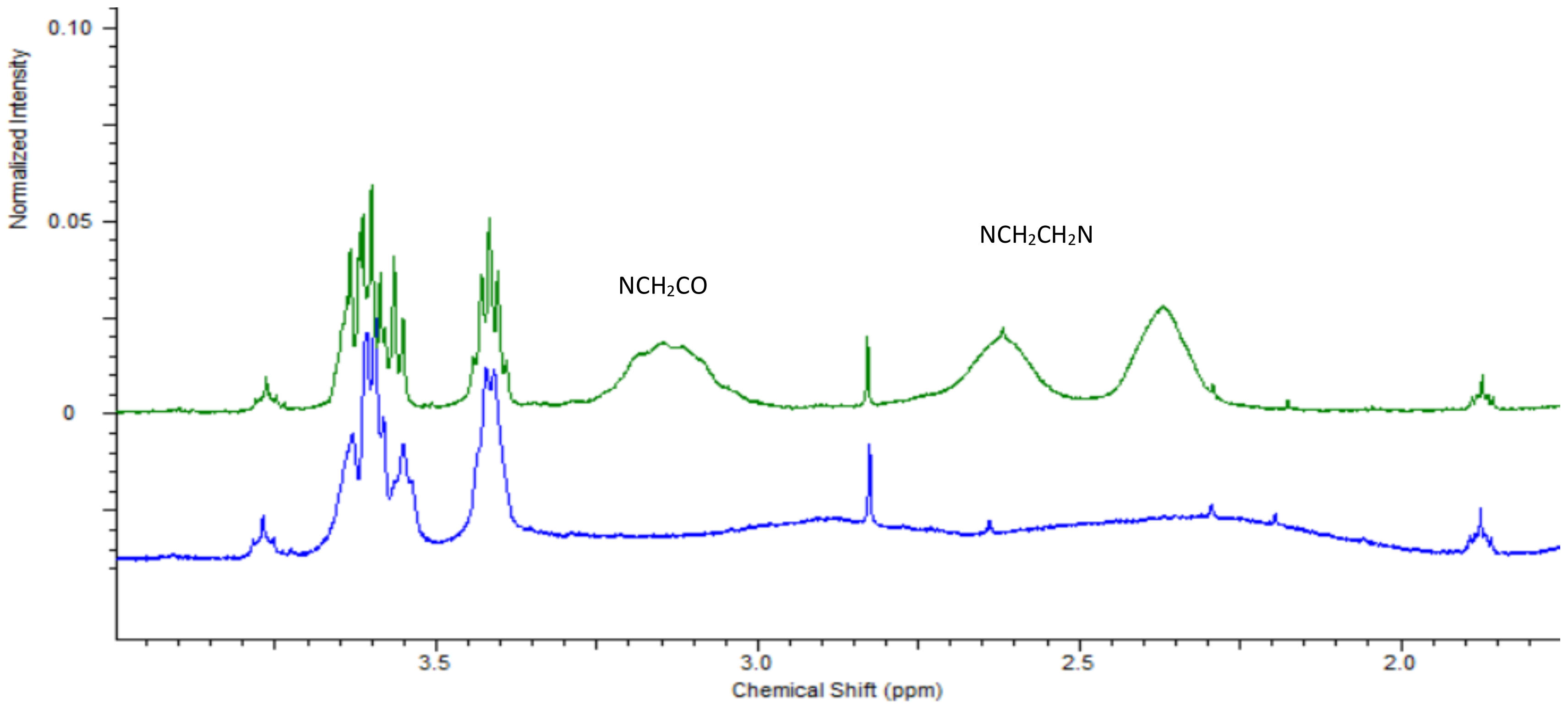

*Comparison of a section of 1H NMR of **3** at 60 ºC (above) and at room temperature (below).*

^{13}C NMR: Tri-*tert*-butyl 2,2',2''-(10-(3,14-dioxo-1-phenyl-2,7,10-trioxa-4,13-diazapentadecan-15-yl)-1,4,7,10-tetraazacyclododecane-1,4,7-triyl)triacetate (**3**)

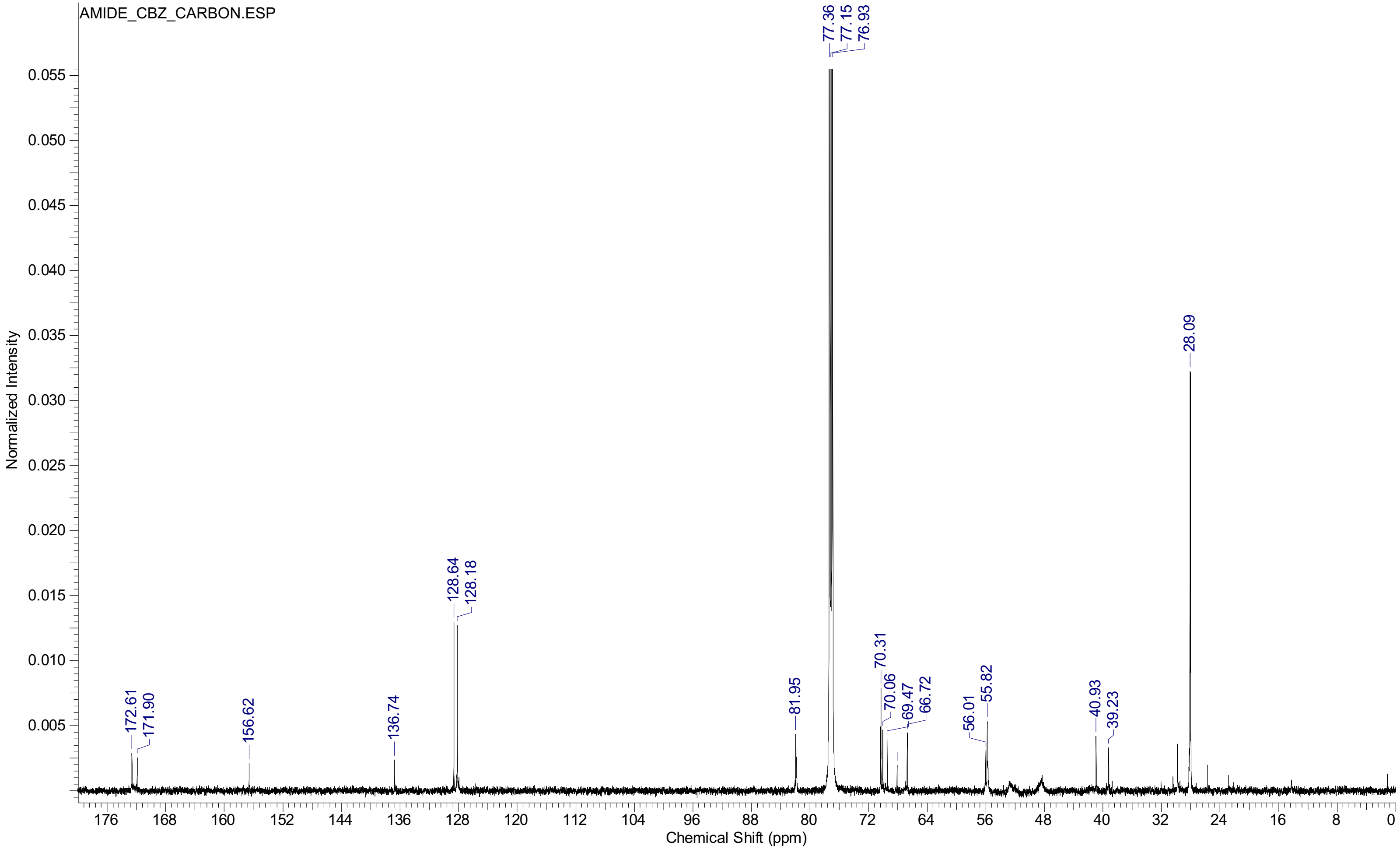

^{1}H NMR: Tri-*tert*-butyl 2,2',2''-(10-(2-((2-(2-(2-aminoethoxy)ethoxy)ethyl)amino)-2-oxoethyl)-1,4,7,10-tetraazacyclododecane-1,4,7-triyl)triacetate (**4**)

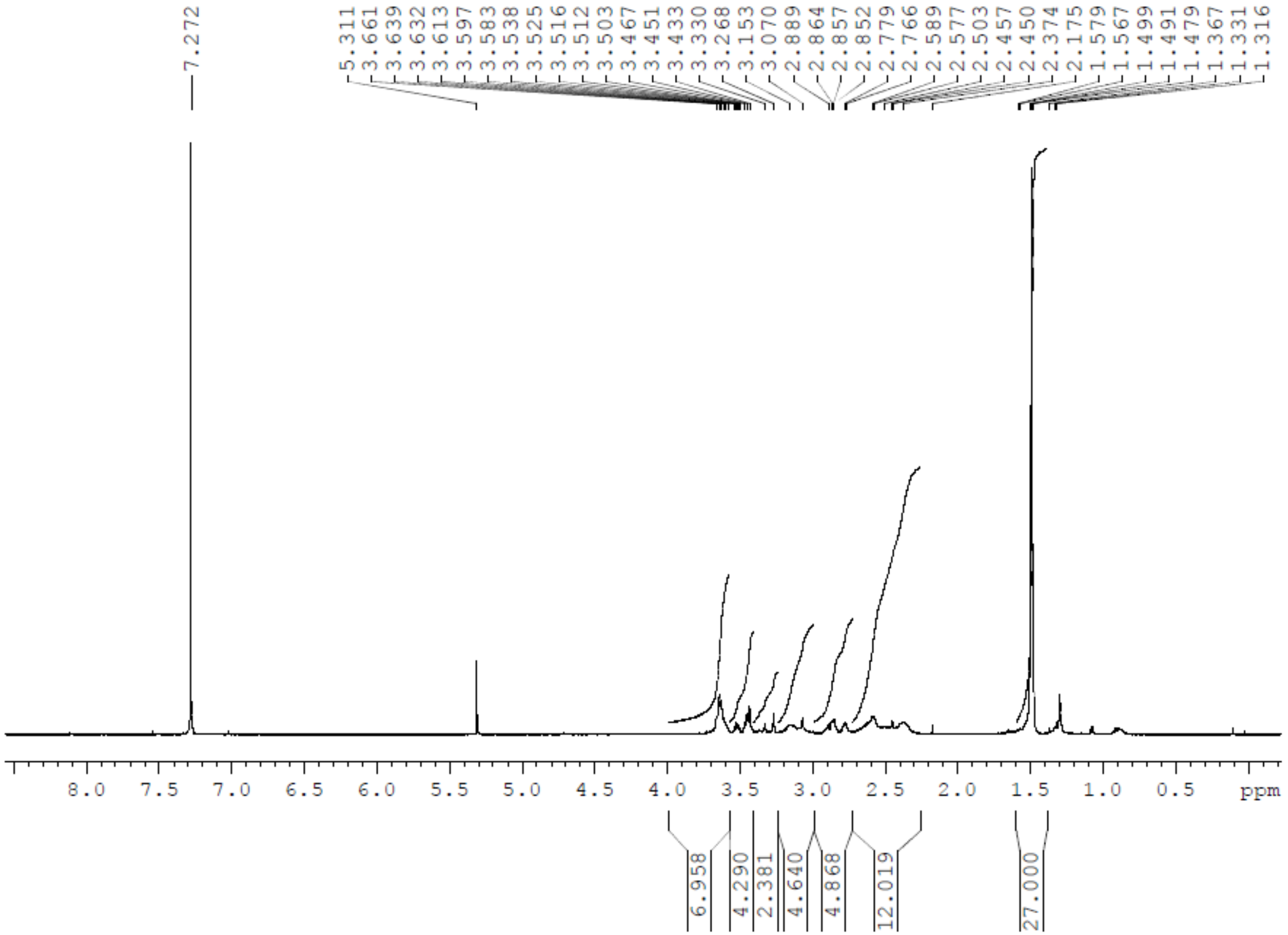

^{13}C NMR: Tri-*tert*-butyl 2,2',2''-(10-(2-((2-(2-(2-aminoethoxy)ethoxy)ethyl)amino)-2-oxoethyl)-1,4,7,10-tetraazacyclododecane-1,4,7-triyl)triacetate (**4**)

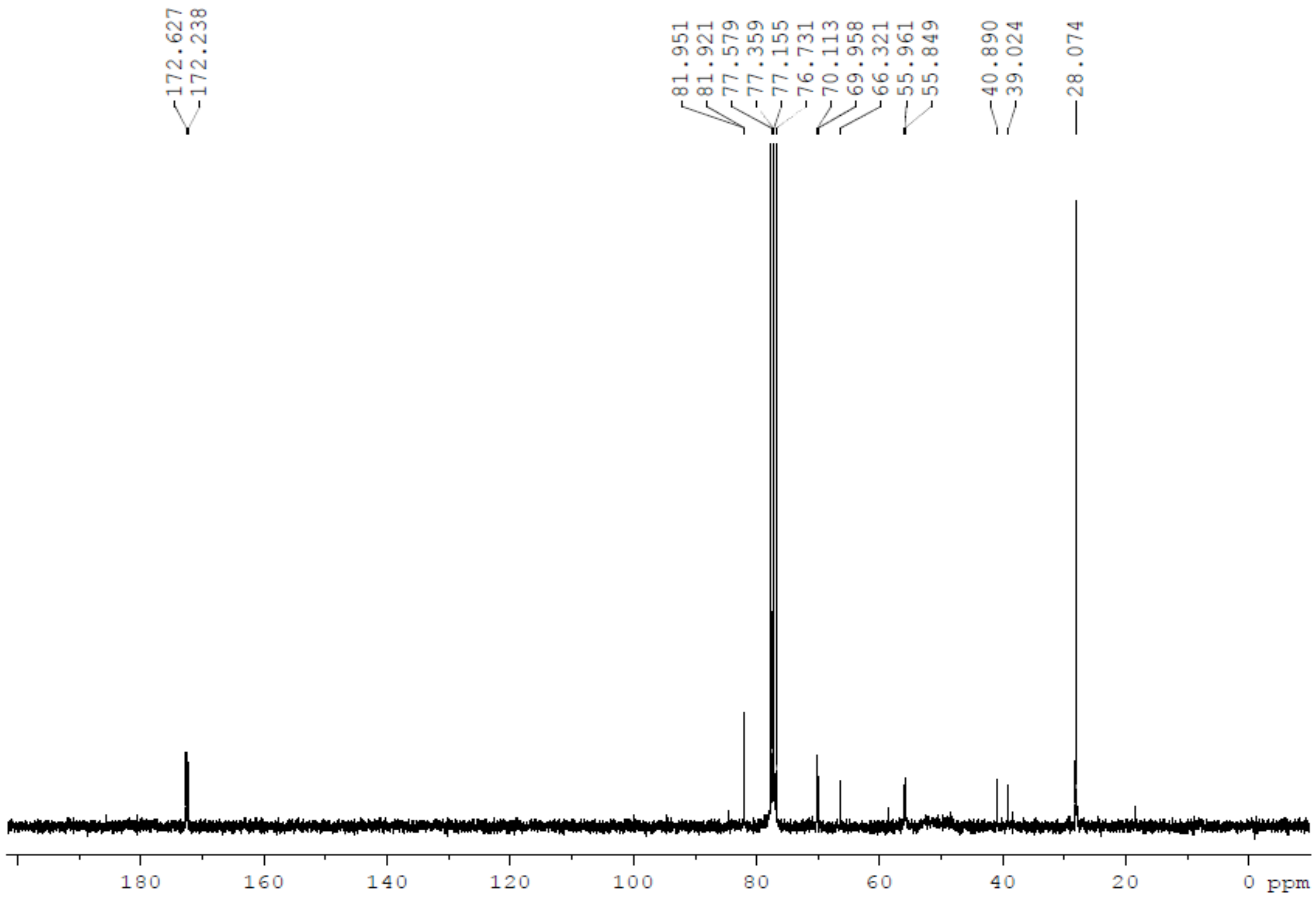

<u>^{1}H NMR: 2,2',2''-(10-(2-((2-(2-(2-aminoethoxy)ethoxy)ethyl)amino)-2-oxoethyl)-1,4,7,10-tetraazacyclododecane-1,4,7-triyl)triacetate (**5**)</u>

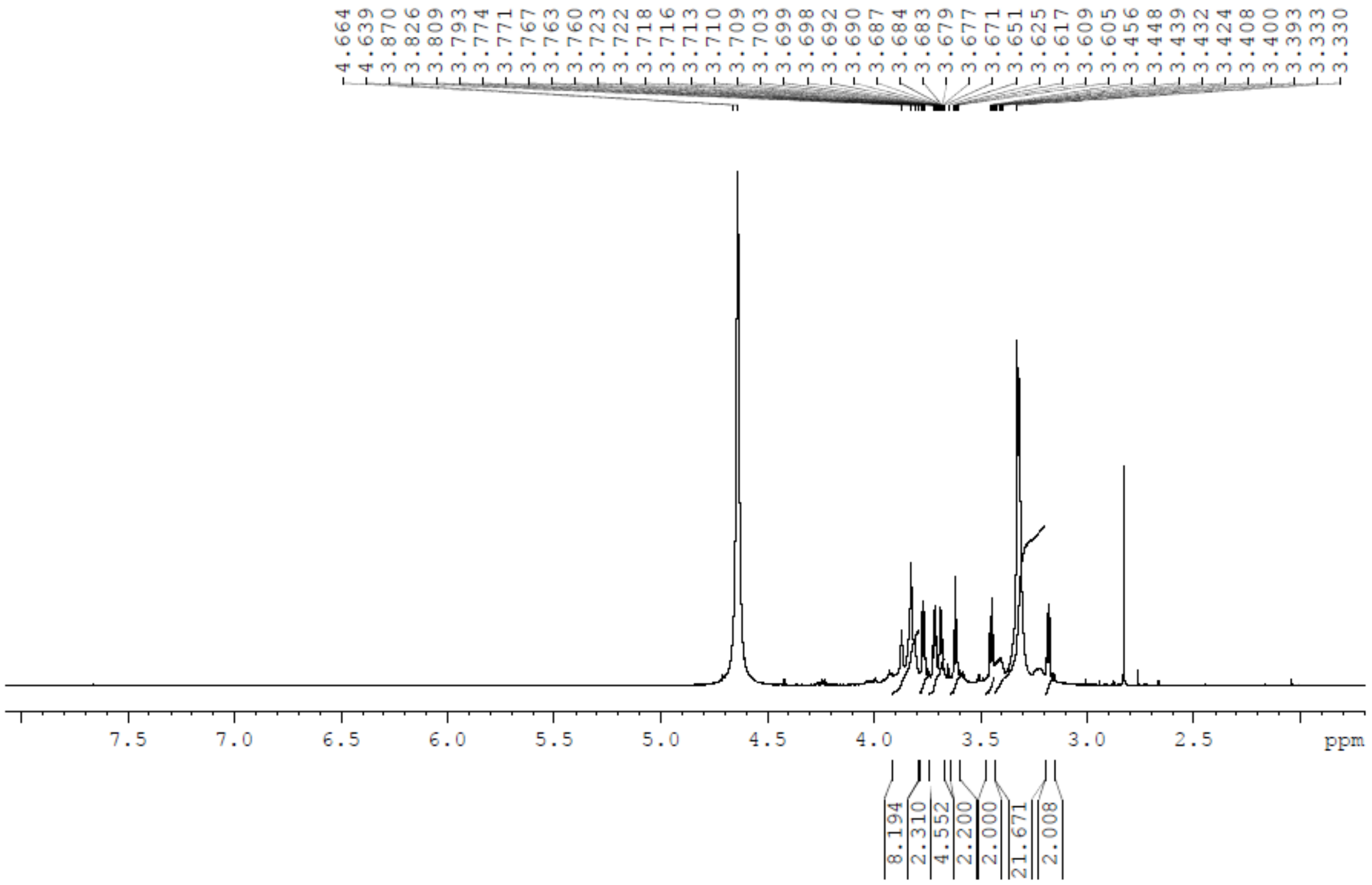

<u>^{1}H NMR: 2,2',2''-(10-(2-((2-(2-(2-aminoethoxy)ethoxy)ethyl)amino)-2-oxoethyl)-1,4,7,10-tetraazacyclododecane-1,4,7-triyl)triacetate (**5**)</u>

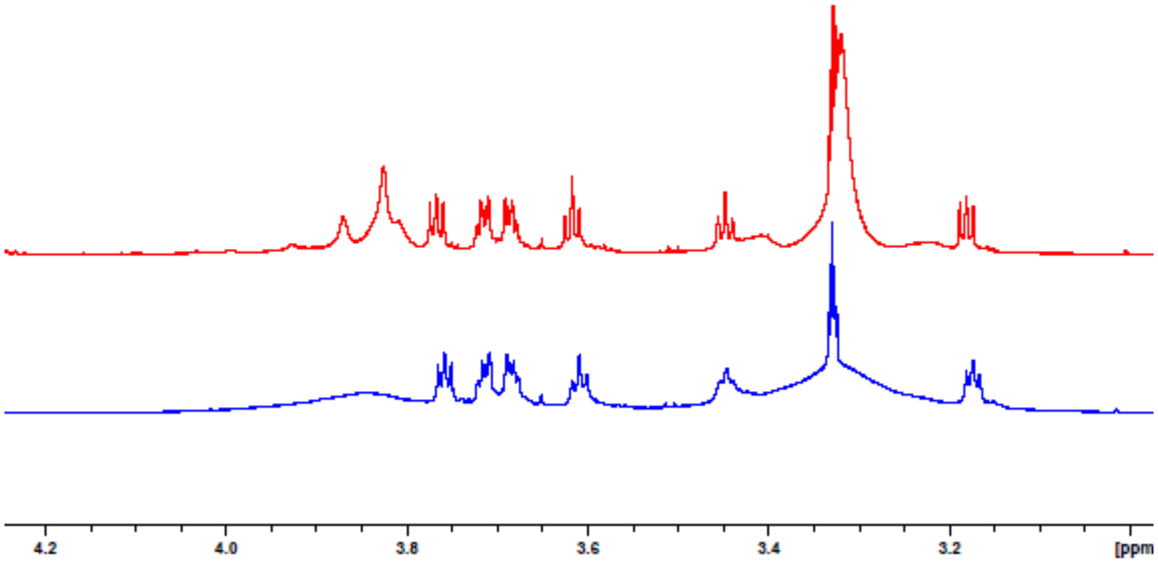

Top spectrum at 60°C, bottom spectrum at rt

^{13}C NMR: 2,2',2''-(10-(2-((2-(2-(2-aminoethoxy)ethoxy)ethyl)amino)-2-oxoethyl)-1,4,7,10-tetraazacyclododecane-1,4,7-triyl)triacetate (**5**)

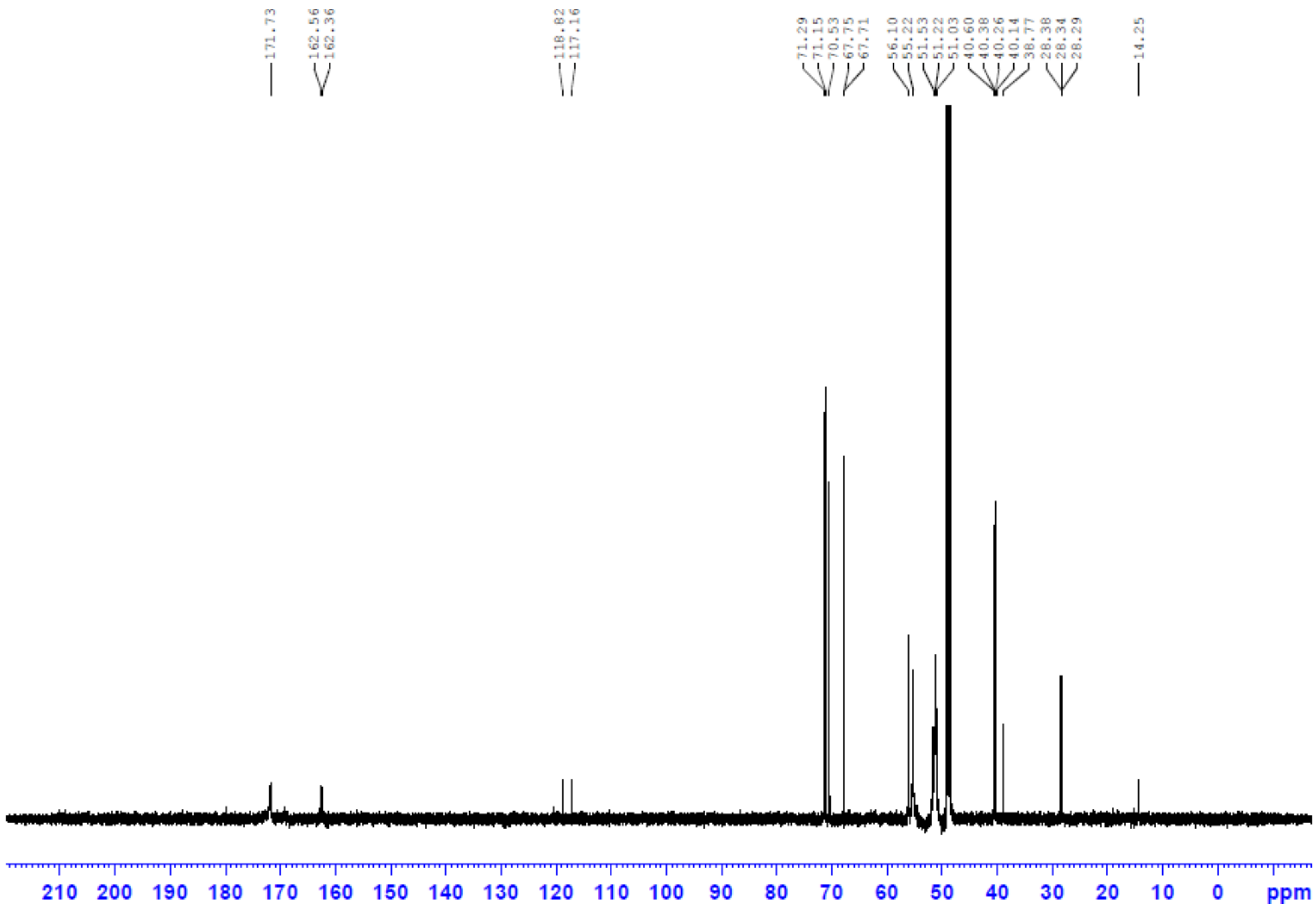

Supplementary Figures

Synthesis and Analysis of Modified SPIONs

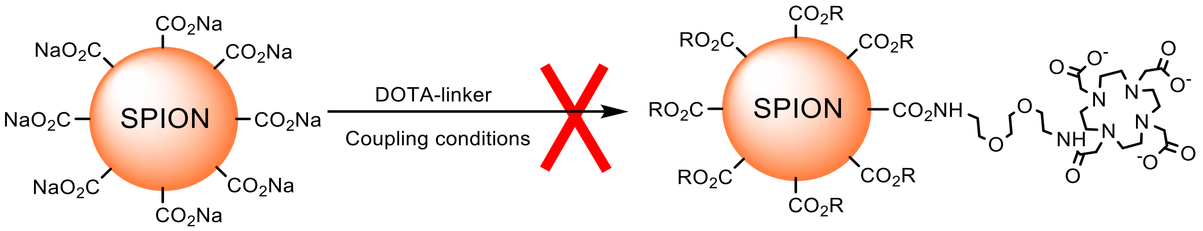

Supplementary FigureS1 – Attempted coupling of DOTA-linker to fluidMag-CT SPIONs.

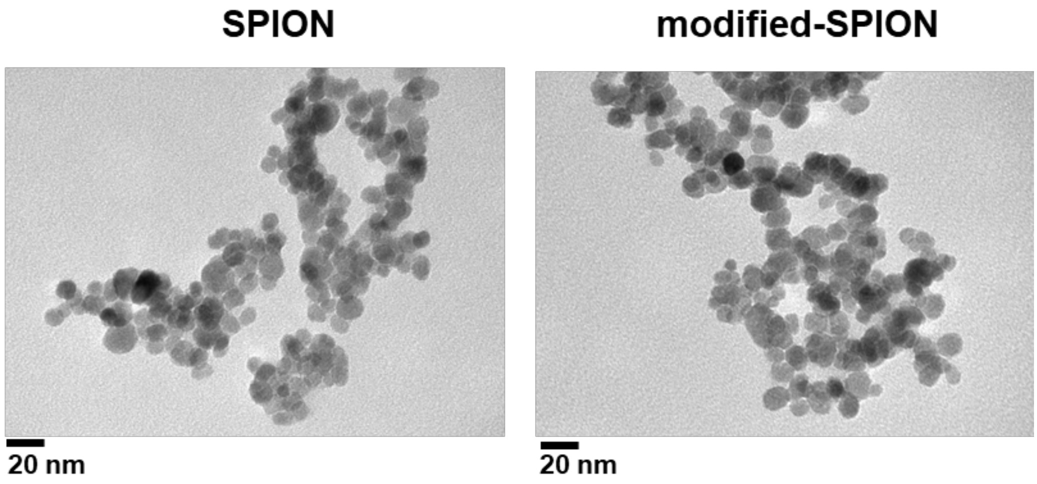

Supplementary FigureS2. TEM images of SPION and modified-SPION in deionised water showing a similar morphological appearance (scale bar = 20 nm).

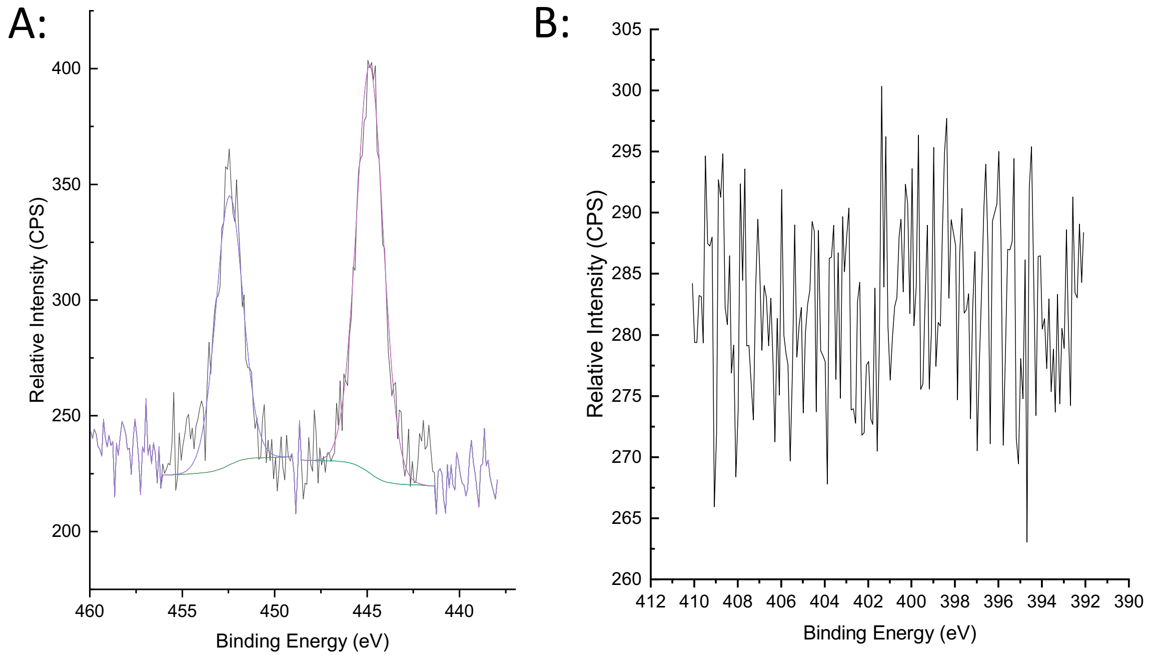

Supplementary FigureS3. High resolution XPS spectra of In-modified- SPION showing: A: In3d (1 environment at 445.1 eV (In3d5/2) and 452.7 eV (In3d3/2)), B: Complete absence of N (1s) peak (surface or after etching).

Stability and Indium Content of Modified SPIONs

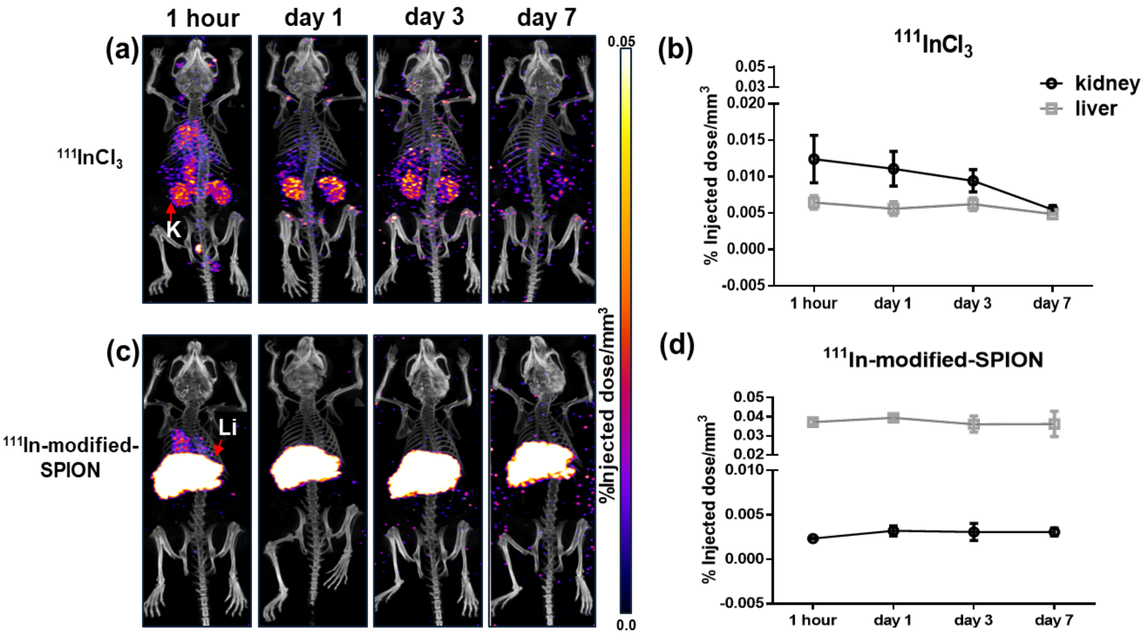

Supplementary FigureS4. *In vivo* validation of ^{111}In-modified-SPION chelation. (a & c) SPECT/CT images at 1 hour, days 1, 3 and 7 after IV injection of free $^{111}InCl_3$ & ^{111}In-modified-SPION (labels: Li = Liver, K = Kidney). (b & d) 3D ROI quantification of SPECT signal in liver and kidney as calculated as %ID/mm^3 after decay correction. Data are shown as mean ± SD, n = 3.

Labelling of ADSCs with ^{111}In-modified-SPION and the Effect on Cell Function

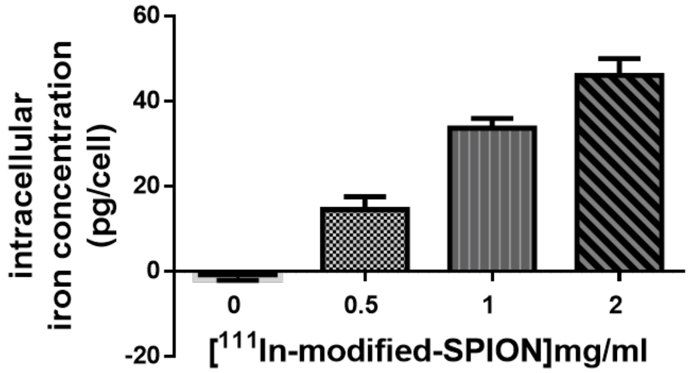

Supplementary FigureS5. Assessment of ^{111}In-modified-SPION uptake in ADSCs using Ferrozine assay. Cells were incubated for 16 hours with ^{111}In-modified-SPIONs at the concentration indicated and intracellular iron uptake was measured. Data are shown as mean ± SD, n = 3.

Labelling of ADSCs with ^{111}In-modified-SPION and the Effect on Cell Function

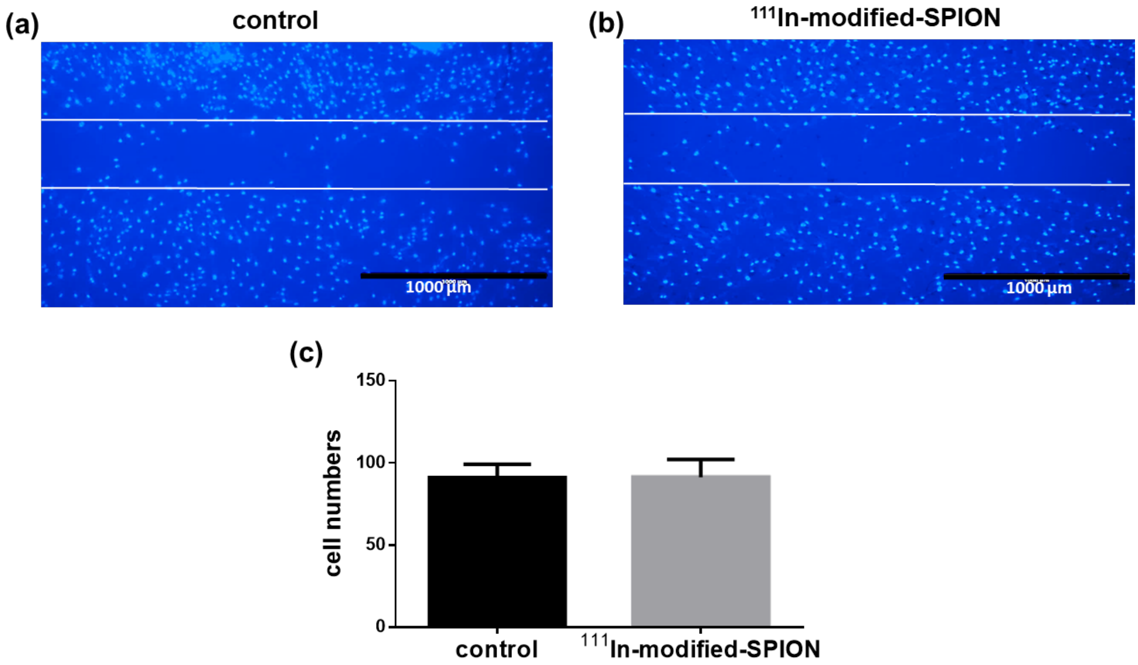

Supplementary FigureS6. The effect of ^{111}In-modified-SPION labelling on ADSC migration. (a) The control cells and (b) ^{111}In-modified-SPION labelled ADSCs were stained with Hoechst33342 for image analysis at 30 hours after removal of culture inserts (scale bar = 1000 µm). (c) The number of cells between the gaps (indicated by white lines) showed no significant difference between the control group and ^{111}In-modified-SPION labelled group. Data are shown as mean ± SD, n = 3.

Labelling of ADSCs with ^{111}In-modified-SPION and the Effect on Cell Function

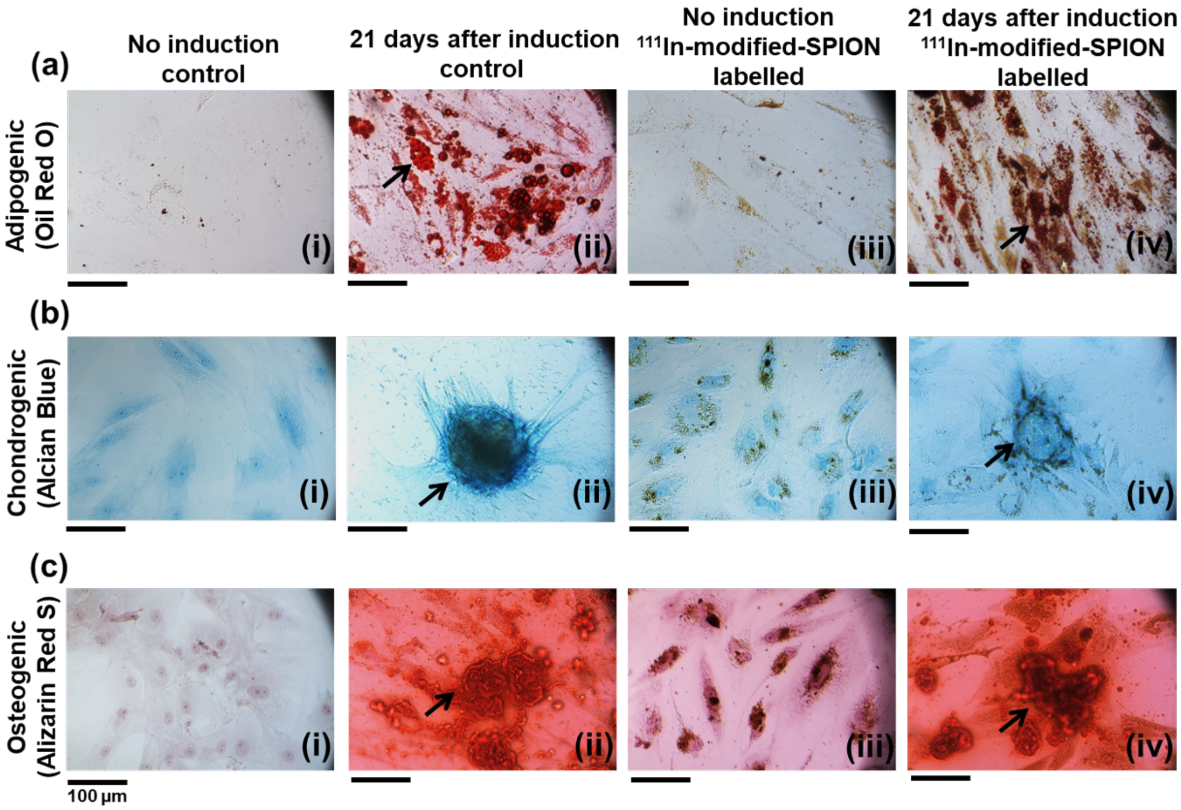

Supplementary FigureS7. Tri-lineage differentiation of control ADSCs and ^{111}In-modified-SPION labelled ADSCs. (a) Oil red o staining for adipogenic differentiation which displays the red coloured oil droplets (indicated by arrows in (ii & iv)). (b) Alcian blue staining for chondrogenic differentiation which displays the blue coloured proteoglycans (indicated by arrows in (ii & iv)). (c) Alizarin red s staining for osteogenic differentiation which displays the red coloured calcium deposits (indicated by arrows in (ii & iv)). Scale bar = 100 µm.

The Effect of Radiolabelling on Cell Viability and Cell Proliferation

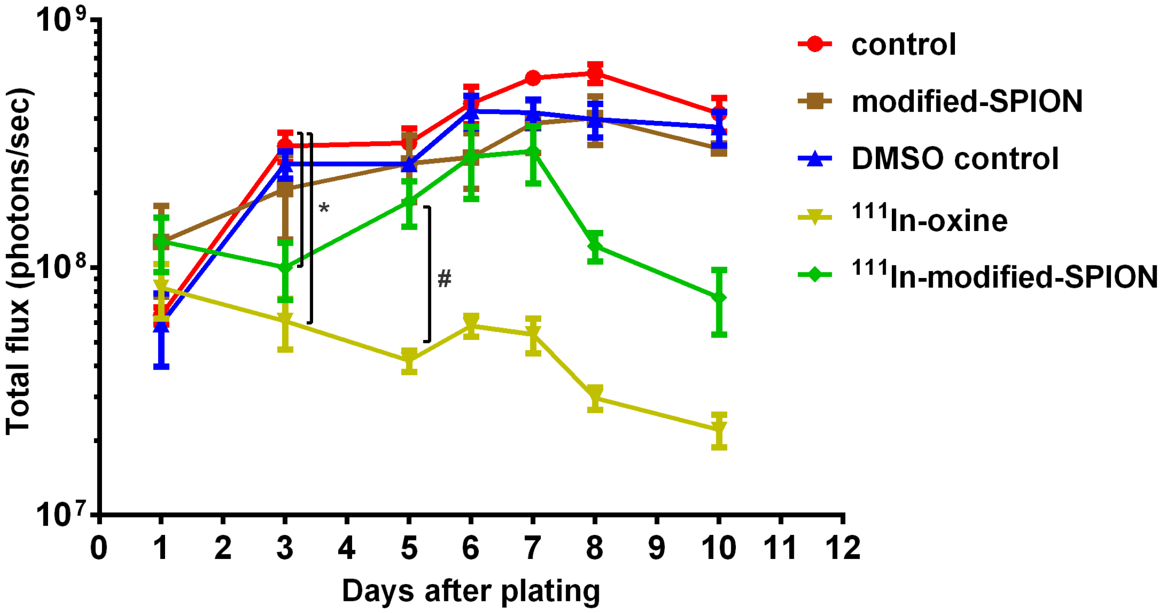

Supplementary FigureS8. The effect of ^{111}In-modified-SPION labelling on cell proliferation. Luciferase-based cell proliferation assay at different time points showing BLI signals (photons/s in log scale) of three non-radiolabelled samples: control cells, cells incubated with DMSO (control vehicle for ^{111}In-oxine) and modified-SPION labelled cells and two radiolabelled samples: cells labelled with ^{111}In-oxine and ^{111}In-modified-SPIONs (* $P < 0.0001$ both radiolabelled cells vs control at 3 days and # $P < 0.0001$ ^{111}In-oxine vs ^{111}In-modified-SPION at 5 days as measured with multiple t test). Data are shown as mean ± SD, n = 3.

Assessment of ^{111}In-modified-SPION Labelled ADSCs Distribution Following IV or IC Injection

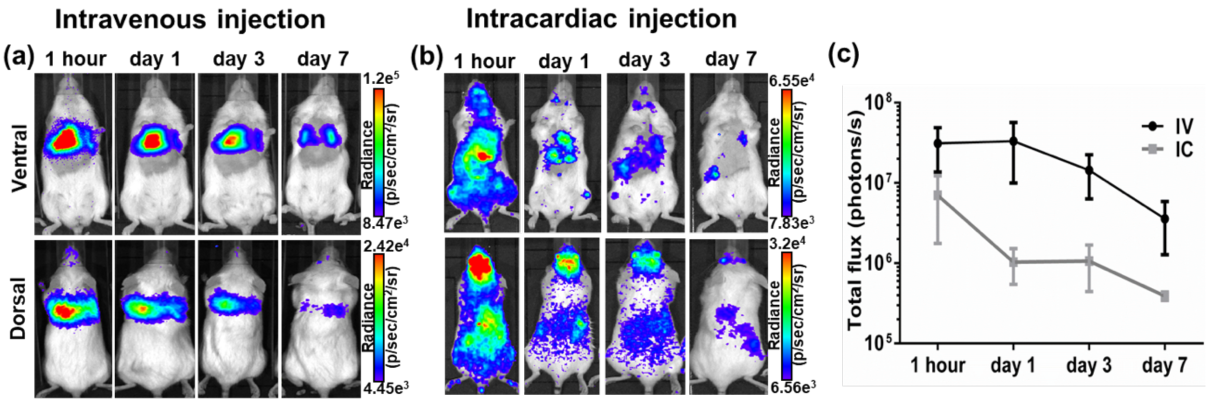

Supplementary FigureS9. BLI images of control ADSC at 1 hour, days 1, 3 and 7 following IV or IC injection. (a, c) BLI signal in the chest and the whole body decreased over time. (b, c) BLI signal in the whole body decreased over time (photons/s in log scale). Data are shown as mean ± SD, n = 4 (IV) & n = 3 (IC).

Assessment of ^{111}In-modified-SPION Labelled ADSCs Distribution Following IV or IC Injection

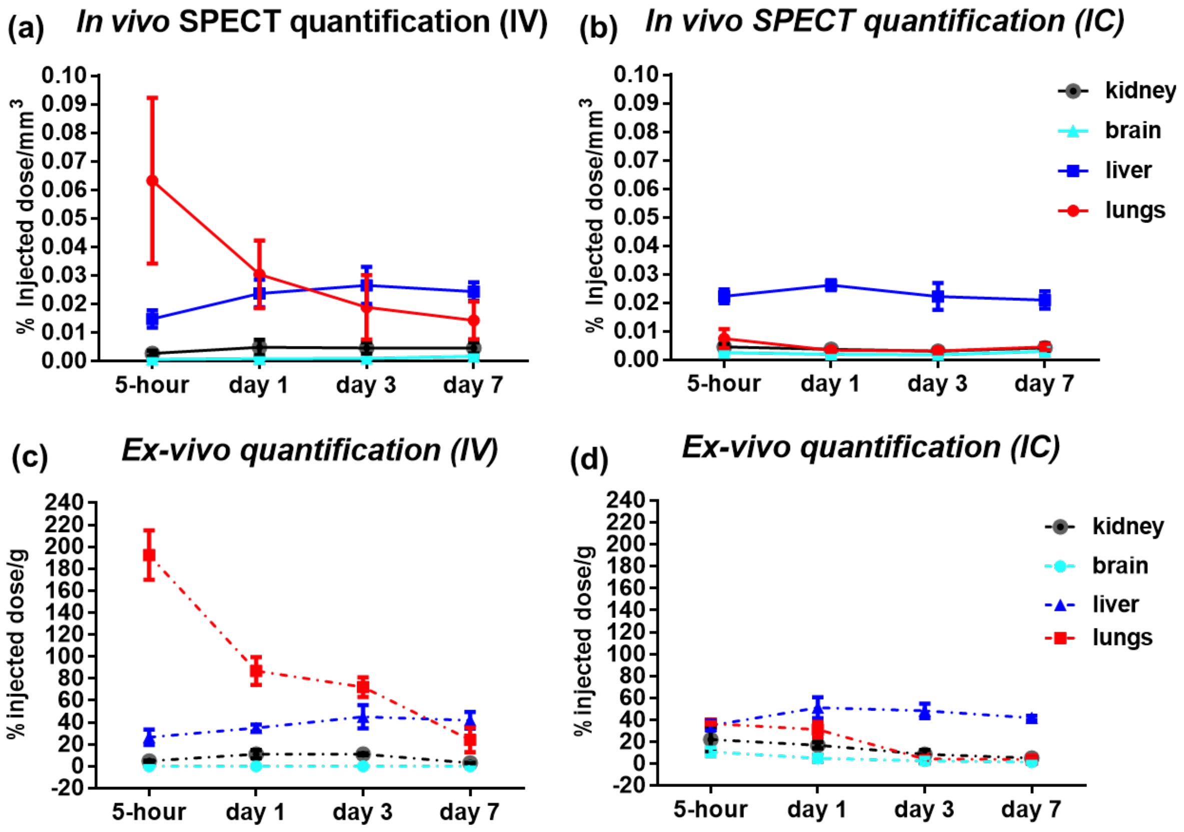

Supplementary FigureS10. *In vivo* and *ex vivo* quantification of ^{111}In-modified-SPION labelled cells uptake in lungs, liver, brain and kidney following IV or IC injection at different time points. (a & b) 3D ROI quantification of *in vivo* SPECT signal in organs after IV & IC injection calculated as %ID/mm^3 after decay correction. (c & d) *Ex vivo* quantification of radioactive distribution in organs after IV and IC injection calculated as %ID/g after decay correction. Data are shown as mean ± SD, n = 5 (*in vivo*) and n = 3 (*ex vivo*).

Assessment of ^{111}In-modified-SPION Labelled ADSCs Distribution Following IV or IC Injection

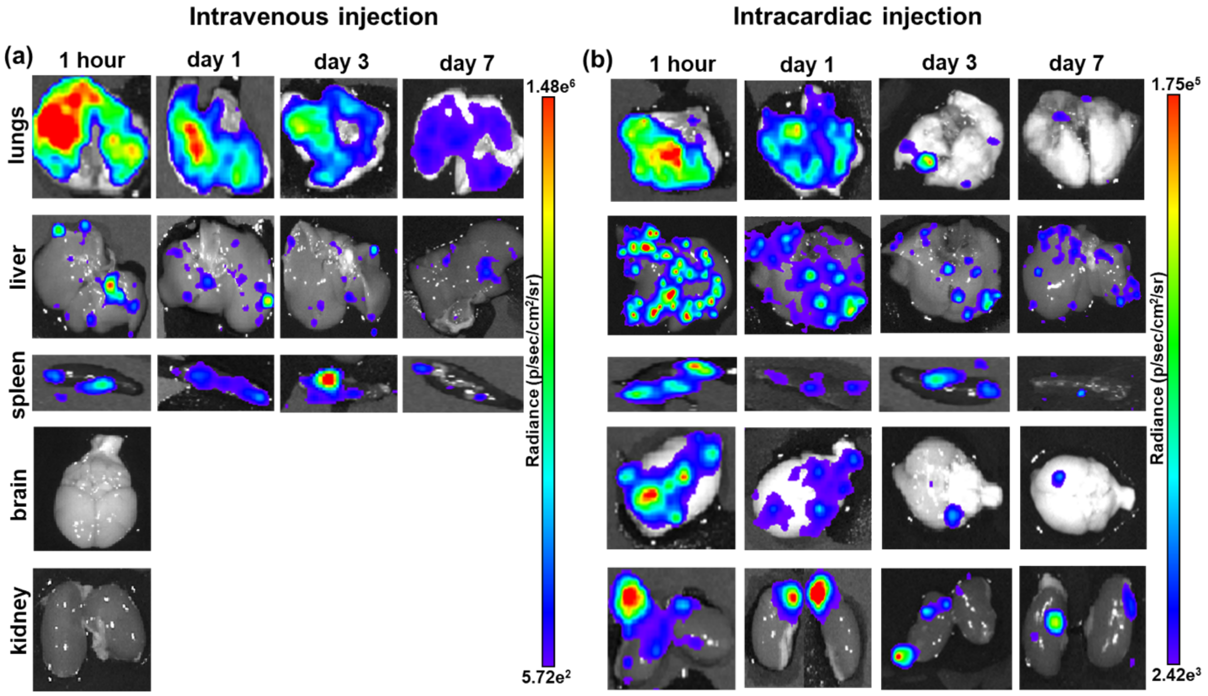

Supplementary FigureS11. *Ex vivo* BLI images of lungs, liver, spleen, brain and kidney at 1 hour, days 1, 3 and 7 after (a) IV and (b) IC injection.

Assessment of ^{111}In-modified-SPION Labelled ADSCs Delivery to Tumour Following IV or IC Injection

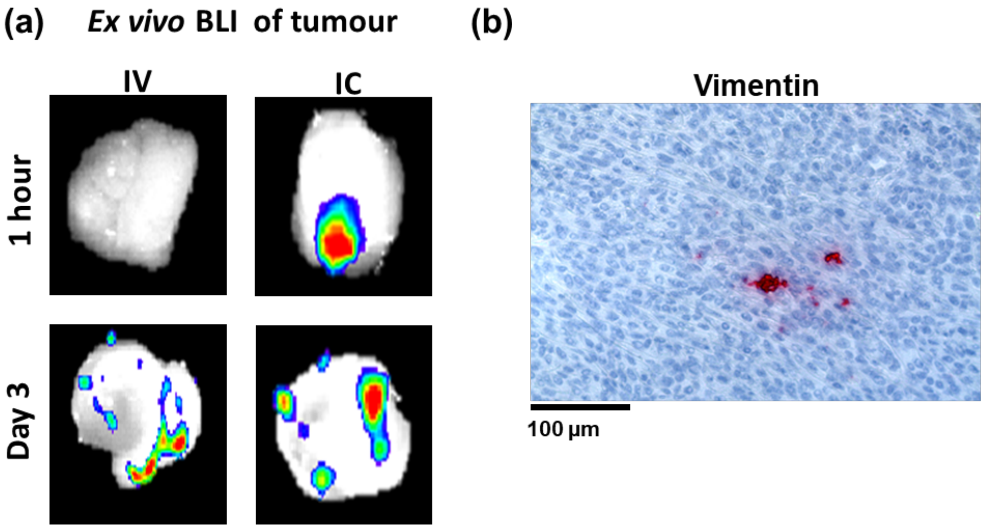

Supplementary FigureS12. ADSC engraftment in tumour following IV and IC injection. (a) *Ex vivo* BLI images of tumour at 1 hour and 3 days after IV or IC injection. (b) Positive vimentin (ADSC) staining of tumour tissue section at day 3 after IC injection (scale bar = 100 µm).

Supplementary Table

Assessment of ^{111}In-modified-SPION Labelled ADSCs Distribution Following IV or IC Injection

Table S1**.** *Ex vivo* quantification of radioactivity distribution (%ID/g) in all organs at 5-hour following IV or IC injection (n = 3 in each group).

**5-hour**	**IV**		**IC**	
	Mean	SD	Mean	SD
**brain**	0.0791	0.015	10.881	3.857
**lungs**	192.442	22.592	36.705	1.007
**liver**	26.405	7.035	34.848	4.377
**kidney**	4.744	0.507	22.205	10.738
**spleen**	4.722	0.923	11.828	2.993
**blood**	3.758	0.348	3.905	0.357
**thyroid & salivary**	0.778	0.092	1.301	0.540
**heart**	0.974	0.204	35.006	2.302
**lymph nodes**	0.597	0.259	0.732	0.471
**stomach**	0.703	0.041	3.132	1.230
**small intestine**	0.976	0.282	10.188	2.521
**caecum**	0.709	0.287	16.788	2.990
**large intestine**	0.646	0.09	5.316	0.588
**muscle**	0.32	0.078	1.526	1.092
**bone**	2.217	0.561	2.517	0.438
**tail**	4.466	4.129	0.629	0.077

Table S2. *Ex vivo* quantification of radioactivity distribution (%ID/g) in all organs at day 1 following IV or IC injection (n = 3 in each group).

**Day 1**	**IV**		**IC**	
	Mean	SD	Mean	SD
**brain**	0.139	0.034	5.030	3.341
**lungs**	86.851	12.696	31.206	7.064
**liver**	34.867	3.085	51.179	9.559
**kidney**	11.181	3.685	17.005	2.950
**spleen**	33.504	2.537	36.354	13.972
**blood**	1.238	0.399	1.315	0.601
**thyroid & salivary**	1.497	0.767	2.145	0.809
**heart**	1.396	0.282	21.261	13.078
**lymph nodes**	0.700	0.163	1.975	1.117
**stomach**	0.947	0.170	2.093	0.532
**small intestine**	2.570	0.761	4.032	1.093
**caecum**	1.849	0.439	3.526	0.982
**large intestine**	1.090	0.234	2.724	0.617
**muscle**	0.429	0.054	0.589	0.116
**bone**	4.061	0.648	3.898	0.865
**tail**	2.902	1.617	0.605	0.255

Table S3. *Ex vivo* quantification of radioactivity distribution (%ID/g) in all organs at day 3 following IV or IC injection (n = 3 in each group).

**Day 3**	**IV**		**IC**	
	Mean	SD	Mean	SD
**brain**	0.137	0.020	2.611	2.751
**lungs**	72.118	9.054	4.468	2.452
**liver**	45.085	10.601	48.321	6.582
**kidney**	11.120	0.915	8.678	3.986
**spleen**	43.785	5.682	29.099	6.343
**blood**	0.491	0.070	0.433	0.112
**thyroid & salivary**	1.433	0.734	2.325	0.961
**heart**	1.325	0.061	9.468	9.189
**lymph nodes**	0.877	0.544	2.540	2.169
**stomach**	1.285	0.126	1.498	0.671
**small intestine**	1.539	0.148	2.314	0.956
**caecum**	1.343	0.385	2.149	1.162
**large intestine**	1.046	0.134	1.359	0.548
**muscle**	0.446	0.074	0.489	0.232
**bone**	5.908	0.263	2.544	0.617
**tail**	2.355	1.321	0.342	0.160

Table S4. *Ex vivo* quantification of radioactivity distribution (%ID/g) in all organs at day 7 following IV or IC injection (n = 3 in each group).

**Day 7**	**IV**		**IC**	
	Mean	SD	Mean	SD
**brain**	0.050	0.005	1.472	0.259
**lungs**	24.000	11.129	4.047	1.477
**liver**	41.945	7.600	41.755	2.057
**kidney**	3.016	0.264	5.156	0.285
**spleen**	18.779	5.541	19.850	2.132
**blood**	0.149	0.024	0.128	0.025
**thyroid & salivary**	0.585	0.156	0.852	0.270
**heart**	0.518	0.106	5.868	2.144
**lymph nodes**	1.536	0.423	1.330	0.119
**stomach**	0.404	0.082	0.787	0.254
**small intestine**	0.353	0.121	1.212	0.371
**caecum**	0.496	0.280	1.184	0.282
**large intestine**	0.449	0.207	0.878	0.212
**muscle**	0.181	0.015	0.267	0.015
**bone**	2.019	0.044	2.151	0.332
**tail**	1.421	0.931	0.297	0.044

Supplementary Information References

1. Mitchell, N.; Kalber, T. L.; Cooper, M. S.; Sunassee, K.; Chalker, S. L.; Shaw, K. P.; Ordidge, K. L.; Badar, A.; Janes, S. M.; Blower, P. J.; Lythgoe, M. F.; Hailes, H. C.; Tabor, A. B., Incorporation of paramagnetic, fluorescent and PET/SPECT contrast agents into liposomes for multimodal imaging. *Biomaterials* **2013,** *34* (4), 1179-92.

2. Meier, J. L.; Mercer, A. C.; Rivera, H.; Burkart, M. D., Synthesis and Evaluation of Bioorthogonal Pantetheine Analogues for in Vivo Protein Modification. *Journal of the American Chemical Society* **2006,** *128* (37), 12174-12184.

3. Green MA, Ke, CY, Leamon CP. Folate mimetics and folate-receptor binding conjugates thereof.*US2011/7875612, B2.*

4. Becker ML, Fang H, Li X, Pan D, Rossin R, Sun X, Taylor J, Turner JL, Welch, MJ, Wooley K L. Cell permeable nanoconjugates of shell-crosslinked knedel (SCK) and peptide nucleic acids (""PNAs"") with uniquely expressed or over-expressed mRNA targeting sequences for early diagnosis and therapy of cancer, US2006/159619, A1.
